# An analytical study of neocartilage from microtia and otoplasty surgical remnants: A possible application for BMP7 in microtia development and regeneration

**DOI:** 10.1371/journal.pone.0234650

**Published:** 2020-06-17

**Authors:** Robin DiFeo Childs, Hitomi Nakao, Noritaka Isogai, Ananth Murthy, William J. Landis

**Affiliations:** 1 Department of Polymer Science, University of Akron, Akron, Ohio, United States of America; 2 Division of Plastic and Reconstructive Surgery, Children’s Hospital Medical Center, Akron, Ohio, United States of America; 3 Department of Plastic and Reconstructive Surgery, Kindai University Medical School, Osaka sayama, Osaka, Japan; Mayo Clinic Minnesota, UNITED STATES

## Abstract

To investigate auricular reconstruction by tissue engineering means, this study compared cartilage regenerated from human chondrocytes obtained from either microtia or normal (conchal) tissues discarded from otoplasties. Isolated cells were expanded in vitro, seeded onto nanopolyglycolic acid (nanoPGA) sheets with or without addition of bone morphogenetic protein-7 (BMP7), and implanted in nude mice for 10 weeks. On specimen harvest, cartilage development was assessed by gross morphology, histology, and RT-qPCR and microarray analyses. Neocartilages from normal and microtia surgical tissues were found equivalent in their dimensions, qualitative degree of proteoglycan and elastic fiber staining, and quantitative gene expression levels of types II and III collagen, elastin, and SOX5. Microarray analysis, applied for the first time for normal and microtia neocartilage comparison, yielded no genes that were statistically significantly different in expression between these two sample groups. These results support use of microtia tissue as a cell source for normal auricular reconstruction. Comparison of normal and microtia cells, each seeded on nanoPGA and supplemented with BMP7 in a slow-release hydrogel, showed statistically significant differences in certain genes identified by microarray analysis. Such differences were also noted in several analyses comparing counterpart seeded cells without BMP7. Summary data suggest a possible application for BMP7 in microtia cartilage regeneration and encourage further studies to elucidate whether such genotypic differences translate to phenotypic characteristics of the human microtic ear. The present work advances understanding relevant to the potential clinical use of microtia surgical remnants as a suitable cell source for tissue engineering of the pinna.

## Introduction

For more than twenty years, the field of auricular tissue engineering has investigated a wide variety of cell sources, polymer scaffolds and growth factors in vitro and in vivo with the hope of making present surgical procedures for auricular repair obsolete [1–5]. Current ear reconstruction implements the use of autologous costal cartilage, Medpor or other prostheses for the auricular framework [6–7]. The surgical process requires advanced training because of its complex and challenging multiple stages [8–12]. Even though many innovations have improved these surgeries, complications and disadvantages for each type of current ear reconstruction still remain [13–15]. Further advancement of auricle reconstruction through tissue engineering approaches requires optimization in the utilization of autologous cartilage biopsies or auricular surgical remnants [16–19].

The most appropriate source of chondrocytes for tissue engineering and regeneration of the ear, its matrix, elastic and mechanical properties is auricular cartilage [20]. Microtia remnants, human auricular, pre-auricular tags and mesenchymal stem cells have also been used as cell sources for seeding ear-shaped polymeric scaffolds [21–25]. The use of auricular chondrocytes isolated from microtia tissue, expanded in culture and grown in the abdominal wall of patients for human autologous ear reconstruction without a scaffold has been reported [26]. Recently, autologous chondrocytes from microtia patients were seeded onto biodegradable scaffolds after expansion in vitro for three months and utilized in auricular reconstructions with patient monitoring for thirty months post-surgery [27].

Controversy persists regarding the use of microtia chondrocytes for tissue engineering because of unknown possible long-term outcomes that may result in a deformed auricle [28]. The current hypotheses for developmental malformations with identified genes and risk factors that result in microtia are numerous [14, 29–30] and microtic ears are as varied in size and shape as the number of patients with the anomaly [31]. For tissue engineering purposes, these normally discarded microtic remnants are typically cleaned of fat and fibrous tissue and then digested to release the chondrocytes from their extracellular matrix (ECM). The isolated cells are then expanded in culture routinely with basic fibroblast growth factor (FGF2), a growth factor known to enhance proliferation by preventing terminal differentiation [32–33]. The expanded microtia-derived cells are seeded onto polymeric scaffolds with or without added growth factors and the same process has also been used for other normal auricular tissues obtained from otoplasties [34]. Bone morphogenetic protein-7 (BMP7; also called osteogenic protein 1, OP-1) exerts well documented effects on ECM secretion of different chondrocyte phenotypes [35–36] and has been used clinically [37] and in tissue engineering paradigms as a growth factor [38–39]. The addition of BMP7 to isolated chondrocytes just prior to implantation may increase the production of ECM and also hinder apoptotic events [40].

The principal purpose of the present study was to examine by several methods, including microarray analysis for the first time, the neocartilages that were generated from cells expanded in vitro from microtia and conchal (normal) surgical tissues and developed 10 weeks in vivo in a nude mouse model. Results of the investigation document the absence of qualitative (morphological and histological) and statistically significant quantitative (RT-qPCR and microarray analyses) differences between the cell types after implantation for 10 weeks in the animals. Such data support the use of microtia cartilage in the tissue-engineering reconstruction of an auricle. When the neocartilages were supplemented with BMP7 present in a slow-release hydrogel, statistically significant changes in the expression levels of certain genes were found by microarray analysis on comparisons between microtia-derived chondrocytes and conchal cells in the presence of BMP7 and between the two cell types with added growth factor and their respective counterpart cell type without the factor. These data suggest a possible application for BMP7 in enhancement of cellular proliferation and ECM production in both normal auricular and microtia cartilage regeneration. While this study lends itself to further investigation, it provides novel evidence for the use of chondrocytes isolated from the normally discarded vestigial ear during microtia reconstruction as a viable source of cells for tissue engineering a replacement auricle in patients undergoing this procedure.

## Materials and methods

### Ethics statement

A protocol for obtaining normally discarded auricular cartilage remnants was approved by an Institutional Review Board at the Children’s Hospital Medical Center of Akron (CHMCA, Akron, OH) and samples were collected with parental-signed consent. Animal care and surgeries were performed in compliance with the Institutional Animal Care and Use Committee (IACUC protocol approved 17-07-163) at the Northeast Ohio Medical University Comparative Medicine Unit (NEOMED CMU, Rootstown, OH) and according to the policies outlined by the National Institutes of Health Guide for the Care and Use of Laboratory Animals (NIH Publications No. 8023, revised 1978). Mice housed at the CMU (NEOMED) were sacrificed by CO_2_ overdose and cervical dislocation following implantation for 10 weeks.

### Isolation of tissues and cells

**Fig 1:**
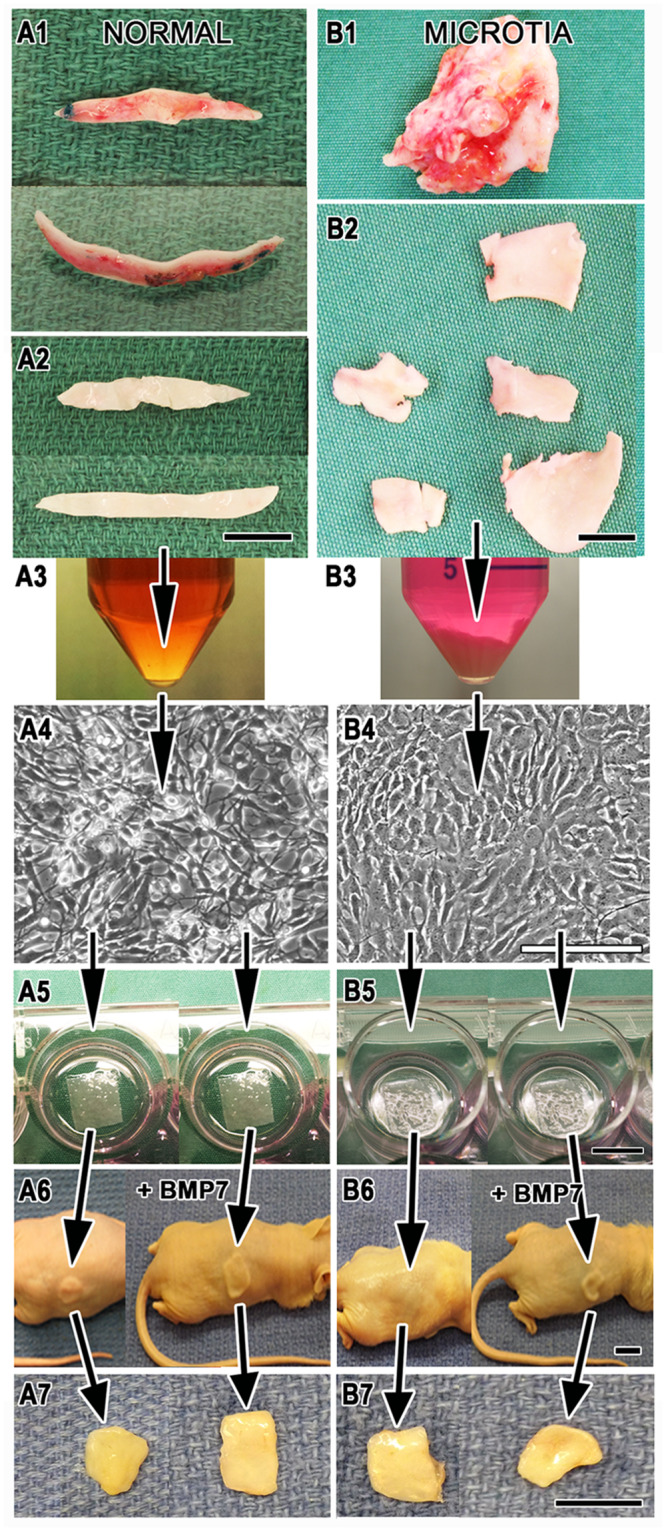
A stepwise depiction of the experimental design of the study showing events from receipt of surgery samples to harvest of tissue-engineered neocartilage from athymic mice. Sample processing is illustrated for auricular chondrocytes isolated from representative human normal (A1-7) and microtia (B1-7) remnant tissues. Remnants were cleaned of fibrous or fatty tissue (A2 and B2), collagenase-digested overnight and filtered (A3 and B3), expanded in vitro (A4 and B4), seeded onto nanoPGA sheets (A5 and B5), implanted subcutaneously with (+) and without BMP7 supplementation (A6 and B6) and harvested after 10 weeks of implantation (A7 and B7). Tissues are highly vascular (red) as obtained from surgery (A1 and B1). A line marks 5 ml in the tube illustrated in B3. A1 and A2 are shown at the same enlargement, B1 and B2 are shown at the same enlargement, and A3-A7 are shown at the same enlargements as their counterpart panels B3-B7. Scale bars = 1 cm (A2, B2, B3, B5, B6, B7) and 200 μm (B4).

Patient demographics for surgical tissues used in the analyses in this study are listed in Table 1. Individuals undergoing an otoplasty provided a source of normal (conchal) auricular cartilage. Microtia cartilage was surgically removed from patients during an auricular reconstruction. Samples stored in cold (4°C), sterile phosphate-buffered saline (PBS, Mediatech, VWR Scientific, Radon, PA) were transferred from the hospital operating room to the laboratory at the University of Akron (Akron, OH). The experimental design for the study is outlined in Fig 1 and described below. Upon arrival at the laboratory, samples were immediately processed using aseptic techniques and following previously published methodologies [33–34]. Briefly, surgical fragments from normal (Fig 1A1) and microtia (Fig 1B1) cartilage were weighed, cleaned of any fibrous or fatty tissue (Fig 1A2 and 1B2) and minced with a sterile scalpel blade into small 3–5 mm pieces. Minced pieces were then digested in 0.3% type II collagenase (Worthington Biochemical, Freehold, NJ) at 37°C for 12 hr (Fig 1A3 and 1B3), and isolated cells were counted and then seeded into flasks [34, 41].

**Table 1:** Patient demographics for each specimen analyzed in this study.

Ref #	RT-qPCRSample	Microarray Sample	Gender	Surgical Age (yrs)	Surgical Tissue	Addition of BMP7
664 R, L	Yes	No	M	5	Microtia	Yes
665 R	Yes	No	M		Microtia	Yes
700 R, L	Yes	Yes	M	14	Normal	Yes
709 L	No	Yes	M		Normal	No
733 L	Yes	Yes	M	10	Normal	Yes
733 R	Yes	Yes	M	6	Microtia	Yes
748 R, L	Yes	Yes	M	4	Microtia	No
749 L	Yes	No	M		Microtia	Yes
796 R	Yes	No	M	9	Microtia	No
797 R*, L	Yes	Yes	M		Microtia	No
798 R, L	Yes	No	M		Microtia	Yes
799 R, L	Yes	Yes	M		Microtia	Yes
813 R	Yes	Yes	M	5	Microtia	No
833 L	Yes	No	F	5	Normal	No
835 L	Yes	Yes	F		Normal	Yes
847 R, L	Yes	No	M	6	Microtia	No
848 L	No	Yes	M		Microtia	Yes
866 R, L	Yes	Yes	F	11	Normal	No
867 R, L	Yes	Yes	F		Normal	No
872 R, L	Yes	No	F		Normal	Yes
873 R, L	Yes	Yes	F		Normal	Yes
908 R	Yes	No	M	13	Normal	Yes
909 R	Yes	No	M		Normal	No
922 R, L	Yes	Yes	M	7	Normal	No
923 R, L	Yes	No	M		Normal	No
924 L	Yes	No	M		Normal	Yes
932 R, L	Yes	Yes	F	7	Microtia	No
933 R, L	Yes	Yes	F		Microtia	Yes

Key: Ref # = study identification number; R = right or L = left side of mouse for implantation; RT-qPCR = reverse transcription-quantitative PCR analysis; F = female; M = male; yrs = years; BMP7 = bone morphogenic protein-7;

* indicates sample failed to bind in microarray analysis. Some patients yielding multiple specimens are noted in the table by Ref # of the same surgical age.

### Cell culture and implantation

Respective donor cells from human normal (Fig 1A4) and microtia (Fig 1B4) auricular cartilage isolated from digested specimens were expanded in culture (37°C, 5% CO_2_) for approximately one week. The complete medium of DMEM/F12 (50:50), 10% fetal bovine serum (Hyclone, Thermo Fisher Scientific, Inc., Waltham, MA), gentamycin, penicillin/streptomycin (VWR Scientific) and primocin (Invivogen, San Diego, CA) was exchanged every other day and supplemented with 10 ng/ml FGF-2 (Biosource, Life Technologies, Grand Island, NY) as previously published [33]. After reaching confluency, cells were trypsinized from the flasks, combined and concentrated to 100 x 10^6^ cells/ml. Nano-polyglycolic acid (nPGA, Gunze Co., Kyoto, Japan) dry and sterile sheets (1 cm x 1 cm x 80 μm in length, width and thickness, respectively) were seeded with 5 x 10^6^ cells (in 50 μl) and subsequently allowed to adsorb or attach for 30 min (37°C, 5% CO_2_) before addition of complete medium (Fig 1A5 and 1B5) [34]. After an additional week in culture, typically two scaffolds were implanted subcutaneously in pockets created to the right and left of the spinal column of 5-to-6-week-old male athymic (nude, *nu/nu*) mice (Envigo, Indianapolis, IN) [34]. Implantation in the mice was carried out for 10 weeks.

Four experimental groups of samples were examined in this study. They were normal isolated auricular chondrocytes cultured without (Group 1) and with BMP7 supplementation (Group 2) and chondrocytes derived from microtia remnants cultured without (Group 3) and with BMP7 addition (Group 4). BMP7 supplementation of Groups 2 and 4 of the cultured cells consisted of the following procedure: 5 μg of BMP7 (Stryker Biotech, Hopkinton, MA) were added to a gelatin-based hydrogel (MedGEL Co., Kyoto, Japan) that allows for the slow release of the bioactive growth factor over 2–3 weeks [42]. A stock solution was freshly prepared by mixing 100 μl of BMP7 (100 μg) with 10 mg of gelatin beads (www.medgel.jp) and incubating the mixture at 37°C for 1 hr. After incubation, the BMP7-loaded gelatin beads were diluted to a volume of 200 μl with sterile PBS (0.5 μg BMP7 in 0.05 mg/ml gelatin beads). One hour before surgery, culture medium was removed from appropriate cell-seeded nanoPGA scaffolds and 10 μl of BMP7-prepared gelatin were applied by pipet to the cell-seeded scaffold constructs (Fig 1A6 and 1B6). Pairs of cell-seeded constructs treated with BMP7 were separately implanted in mice from animals implanted with constructs without BMP7.

### Construct retrieval and gross morphology

Mice housed at the CMU (NEOMED) were sacrificed by CO_2_ overdose and cervical dislocation following implantation for 10 weeks. Auricular cell-seeded scaffolds were harvested typically from both sides (R and L, Table 1) of the mice (Fig 1A7 and 1B7), photographed and stored immediately in either RNA*later* (Ambion, Thermo Fisher Scientific, Inc.) at 4°C or 10% neutral buffered formalin (NBF) at room temperature. After 24 hr at 4°C, samples preserved in RNA*later* were frozen at -80°C for subsequent gene expression and microarray analysis [34]. Samples in NBF were examined by histological methods.

### Histology

Samples retrieved from NBF were dehydrated in graded ethanols, embedded in paraffin, and cut on a microtome into serial sections 5 μm thick. Deparaffinized and rehydrated sections were stained with Safranin-O red for detection of proteoglycans and Verhoeff solution for identification of elastic fibers [34].

### Gene expression analyses

Based on detailed procedures from this laboratory [34, 40], total RNA was isolated from samples (n = 10 for each group, Table 1) initially frozen and stored as noted above, then DNase-treated as part of the PicoPure column purification (Arcturus, Thermo Fisher Scientific, Inc.). RNA quantity and quality were assessed by spectrophotometric means [33–34]. Purified RNA was reverse-transcribed to cDNA as previously described [33–34].

An ABI Prism 7500 Sequence Detector (Applied Biosystems, Thermo Fisher Scientific, Inc.) was used for quantitative PCR, and human-specific TaqMan^®^primers of six genes selected for investigation, type II collagen (Hs00264051_m1), type III collagen (Hs00943809_m1), elastin (Hs00355783_m1), SOX5 (Hs00374709_m1), 18S rRNA (4352930E) and large ribosomal protein (P0, 4326314E), were purchased (Applied Biosystems). 18S rRNA and P0 were utilized as reference genes for the study. The PCR reaction was initiated for 10 minutes at 95°C and cycled 40 times sequentially at 95°C for 15 s and 60°C for 60 s following the recommended parameters of the manufacturer. Levels of expressed genes were measured by the standard curve methodology and normalized to P0 according to standardized protocols (http://www.invitrogen.com/site/us/en/home/Products-and-services/Applications/PCR/real-time-pcr/qpcr-education/absolute-vs-relative-quantification-for-qpcr.html). Quality of sample cDNA was determined by quantity generated from 18S rRNA PCR amplification and differences from average numbers of the sample group. Those samples of poor quality based on these results were removed from the study.

### Microarray analysis

Specimens stored at -80°C for microarray analysis were separately ground to powders under liquid nitrogen in a Spex 6870 freezer/grinder mill (Spex, Inc., Metuchen, NJ) [34]. Each powder was transferred to a sterile tube containing 2–3 ml of TRI Reagent^®^ (Molecular Research Center, Inc., Cincinnati, OH) and total RNA was isolated according to the protocol of the manufacturer. Purification of total RNA was accomplished with a PicoPure kit (Arcturus, Thermo Fisher Scientific, Inc.) [33–34]. Purified RNA for microarray analysis was shipped on dry ice to the Biomedical Genomics Core of The Research Institute at Nationwide Children’s Hospital, Columbus, OH, an Agilent (Agilent Technologies, Santa Clara, CA)-certified service provider [43]. Two separate microarrays were performed using the SurePrint G3 Human GE 8X60 K (Agilent Technologies). One microarray consisted of a comparison of gene expression levels between neocartilage generated from microtic (n = 4) and normal conchal (n = 4) surgical remnants after 10 weeks of implantation of the specimens (Table 1). A second microarray comparison was conducted over the same 10-week implantation period with microtia (n = 4) and normal (n = 4) chondrocyte-seeded nanoPGA scaffolds supplemented with BMP7 (Table 1) in the gelatin slow-release delivery system described above.

Analyses of the four experimental sample groups by the Biomedical Genomics Core identified significantly differentially expressed genes applying two well validated and commonly utilized statistical approaches, significance analysis of microarrays (SAM) and adjusted p-value (http://genomics.nchresearch.org). SAM calculates a q-value that is more reliable then p-value and is based on the parameter of false discovery rate (FDR) (See S1 File) [44]. A highly strict value of 10% FDR was utilized initially for microarray analysis, but it yielded very few significantly differentially expressed genes in the sample groups. A less stringent 25% FDR was subsequently chosen by the Biomedical Genomics Core for analysis in order to increase the probability of identifying genes naturally highly variable or weak or rapidly changing in expression, as is typical of biological tissues (See S1 File). Summary reports of all microarray data and statistically significantly expressed genes found in the groups examined utilizing 25% FDR and determined by the Biomedical Genomics Core at Nationwide Children’s Hospital are deposited in the NCBI Gene Expression Omnibus [45] and are accessible through GEO Series accession number GSE136198 (https://www.ncbi.nlm.nih.gov/geo/query/acc.cgi?acc=GSE136198).

### Statistical analyses

Measurements of specimen width, length and thickness utilized digital images and Adobe Photoshop CS6 software (Adobe Systems Inc., San Jose, CA) as detailed previously [34]. Specimen dimension average ± SD and trendline equations with their corresponding R^2^ (coefficient of determination) values were graphically plotted and tabulated with Microsoft Excel 2016 (Microsoft Corp., Redmond, WA).

Data were plotted with Microsoft Excel 2016 (Microsoft Corp.) for each of the two neocartilage tissue types with and without BMP7 supplementation, and averages were reported for types II and III collagen, elastin, and SOX5, the principal genes of interest, normalized to P0 with their respective standard errors of the mean [33–34]. Trendline equations with their corresponding R^2^ values for the four genes of interest were tabulated with Microsoft Excel 2016 (Microsoft Corp.), also comparing the two neocartilage tissue types with and without BMP7 supplementation. Statistical analyses of the gene expression measures by RT-qPCR for the four sample groups were performed using ANOVA and a Bonferroni multiple comparisons test (IBM SPSS Statistics 20, Chicago, IL). Statistical significance for all analyses was defined as p ≤ 0.05.

## Results

**Fig 2:**
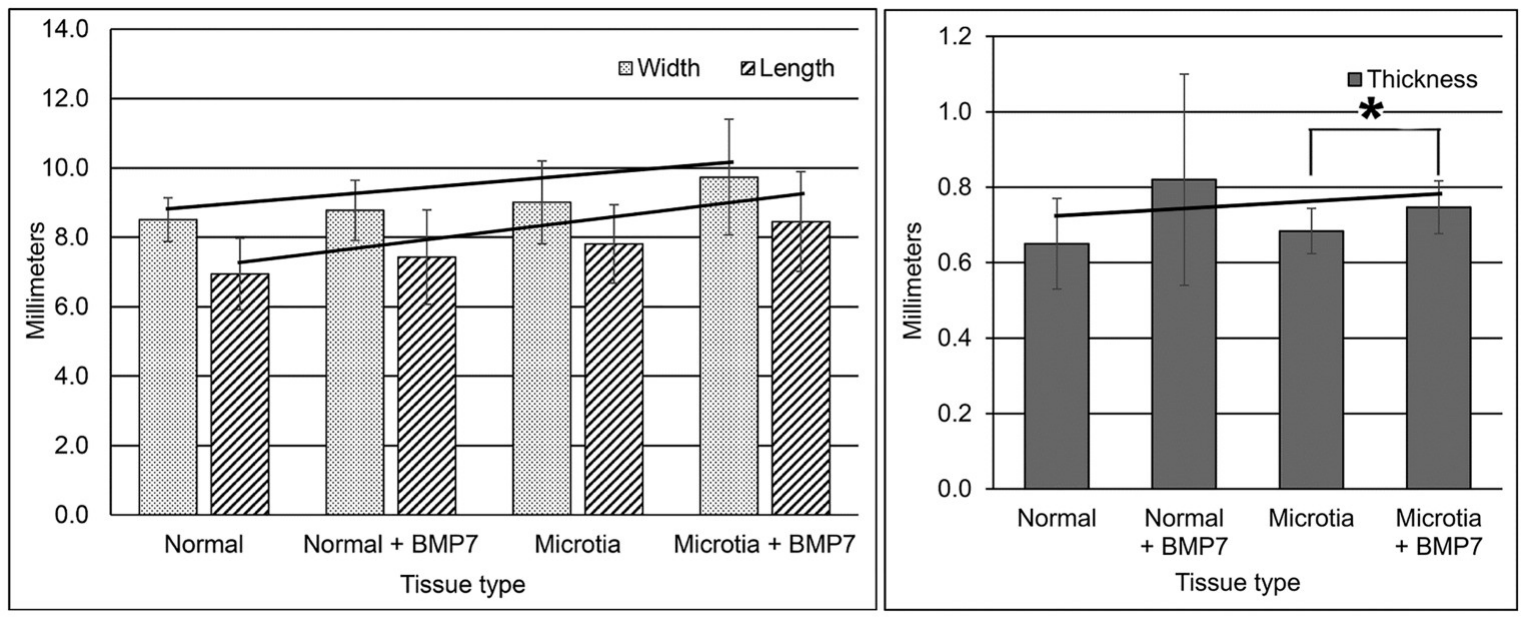
Morphometric analysis of the width, length and thickness of auricular cartilage samples retrieved following implantation for 10 weeks in athymic mice.

Fig 1 as described briefly above presents a schematic of the experimental protocol for the native surgical tissues, including their processing and then retrieval from nude mice after 10 weeks of implantation. Morphometric analyses of width, length and thickness of harvested neocartilage samples are presented as mean values for each of four sample groups with respective standard deviations in Fig 2. Representative trendlines are depicted in the comparison of all four groups for width, length and thickness in Fig 2 with correlated R^2^ values (Table 2). Additional trendline values calculated for comparison of the individual groups with and without addition of BMP7 are tabulated in Table 2. R^2^ data were equal to or approached the value of 1 for all trendline calculations except thickness measures for the four groups. Microtia and normal groups whose cells were absent of BMP7 were not statistically significantly different in dimensions when compared (Fig 2). Microtia and normal groups whose cells were supplemented with BMP7 when compared were also not statistically significantly different (Fig 2). BMP7-treated cell/scaffold constructs showed a trend toward increasing width and length compared to their non-treated counterparts (Fig 2 and Table 2). The thickness of these same growth factor-enhanced neocartilages appeared greater than the thickness of their respective non-treated samples, and microtia with BMP7 when compared to microtia without BMP7 was statistically significantly increased (Fig 2).

**Table 2:** Trendline equations, R^2^ values and trendline slopes for specimen width, length and thickness measures graphically presented in Fig 2. Calculations were made for trendlines between all groups (four points) and between each biopsy tissue type with and without BMP7 (two points) for each dimension of interest.

	Parameter	All groups	Normal and Normal + BMP7	Microtia and Microtia + BMP7
Width	Line equation	y = 0.434x + 7.858	y = 0.192x + 8.226	y = 0.723x + 8.287
	R^2^	0.929	1	1
	Slope	Positive	Positive	Positive
Length	Line equation	y = 0.641x + 5.888	y = 0.640x + 5.890	y = 0.640x + 7.170
	R^2^	1	1	1
	Slope	Positive	Positive	Positive
Thickness	Line equation	y = 0.013x + 0.682	y = 0.070x + 0.610	y = 0.070x + 0.610
	R^2^	0.203	1	1
	Slope	Slight positive	Slight positive	Slight positive

Parameter mean values with respective standard deviations were plotted for width and length (left panel) and thickness (right panel) for control normal (conchal) and microtia neocartilages with and without BMP7 supplementation. There was a trend toward slightly increased width, length and thickness when BMP7-enhanced groups were compared to their respective groups without BMP7 (Table 2). A statistically significant difference (*) was noted only in the thickness comparison of microtia with and without BMP7 addition (p < 0.05). N = 13 for each of the four groups of samples. Error bars represent standard deviation (SD) values of respective sample dimensions.

**Fig 3:**
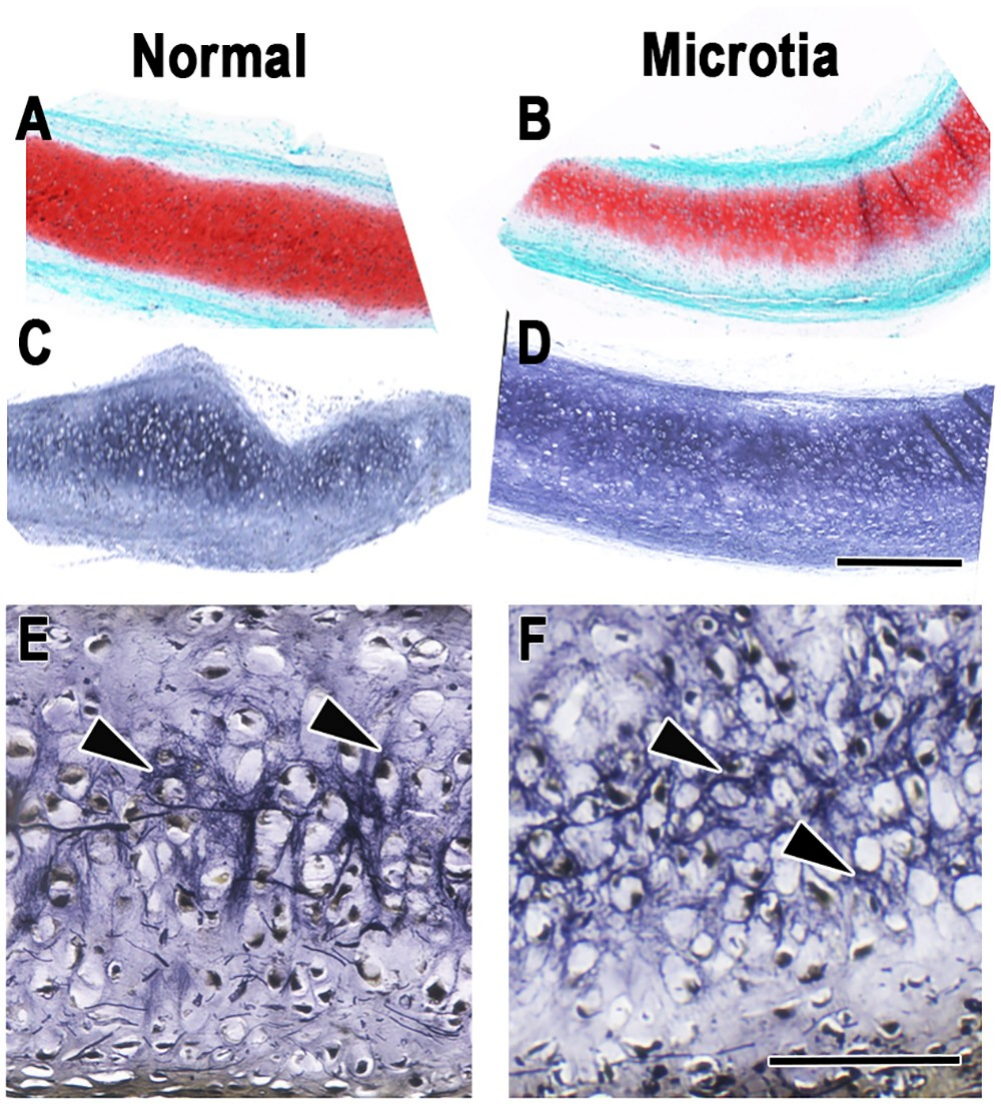
Representative histology of regenerated human microtia and normal auricular cartilage harvested following implantation for 10 weeks in nude mice. Safranin-O red staining reveals secreted proteoglycans from auricular chondrocytes in normal (A) and microtia (B) neocartilages. Verhoeff staining of normal (C) and microtia (D) sections shows the presence of elastin, and elastic fibers (arrowheads) are visible upon higher magnification of normal (E) and microtia (F) neocartilages. Scale bars = 0.5 mm (D and A-C at the same enlargement), 100 μm (F and E at the same enlargement).

Fig 3 illustrates representative histological sections of neocartilage generated from either normal (Fig 3A, 3C and 3E) or microtia surgical remnants (Fig 3B, 3D and 3F) after 10 weeks of implantation in nude mice. Proteoglycans (Fig 3A and 3B) appeared as a richly red-stained matrix surrounding chondrocytes residing in their lacunae and typical of a cartilage phenotype. Elastic fibers in the tissues were evident by Verhoeff staining of normal and microtia (Fig 3C and 3D, respectively) sections and were distinctive upon higher magnification (Fig 3E and 3F). Both neocartilages appeared grossly similar in appearance to each other as well as to classical Safranin-O red- and Verhoeff-stained histological sections from native auricular cartilage [34].

**Fig 4:**
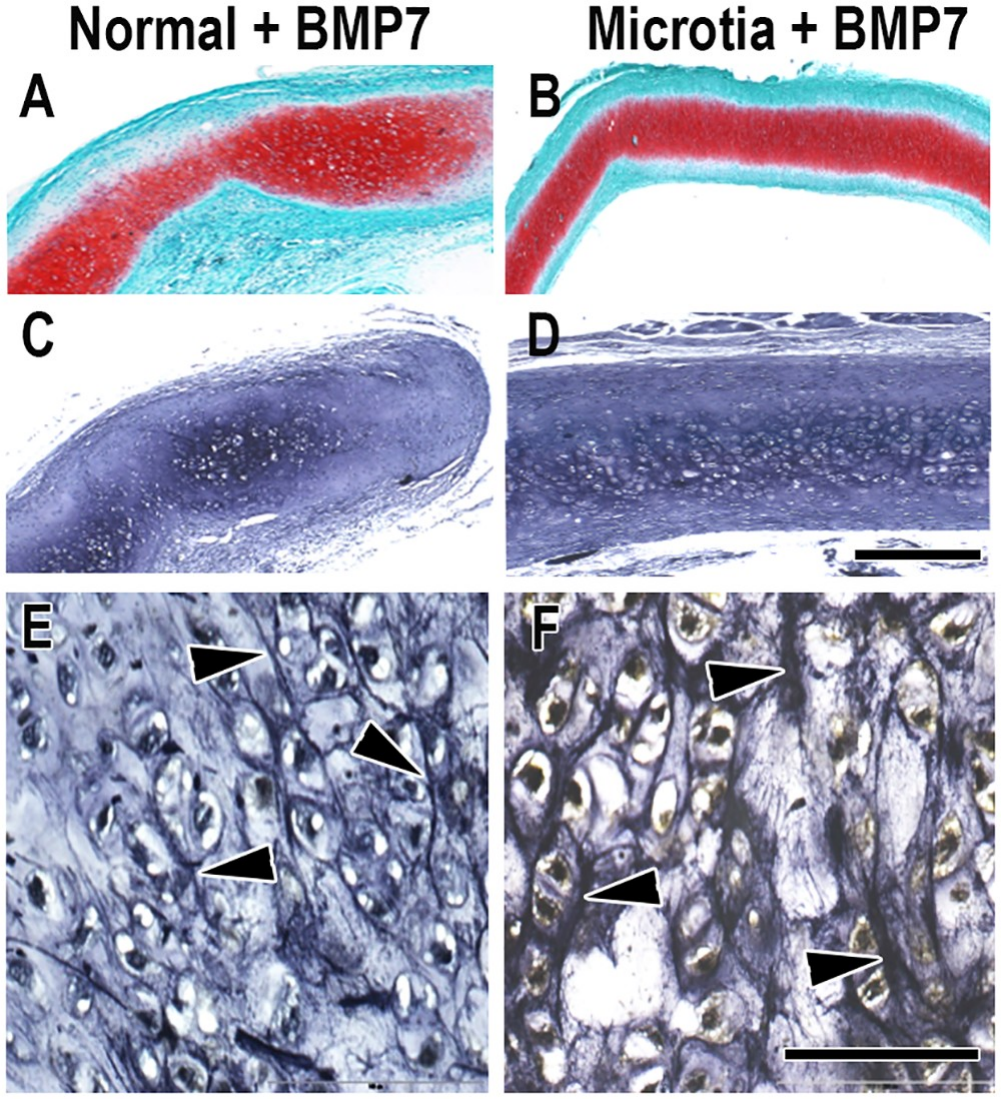
Representative histology of regenerated human microtia and normal auricular cartilage supplemented with BMP7 and harvested following implantation for 10 weeks in nude mice. Safranin-O red staining reveals secreted proteoglycans from auricular chondrocytes in normal (A) and microtia (B) neocartilages. Verhoeff staining of normal (C) and microtia (D) sections shows the presence of elastin, and elastic fibers (arrowheads) are visible upon higher magnification of normal (E) and microtia (F) neocartilages. Scale bars = 0.5 mm (D and A-C at the same enlargement), 100 μm (F and E at the same enlargement).

Fig 4 presents histology of neocartilage samples whose cells were pretreated with BMP7 prior to implantation in nude mice for 10 weeks. Normal neocartilage developed following supplementation with BMP7 (Fig 4A, 4C and 4E) and neocartilage obtained from microtic cells supplemented with BMP7 (Fig 4B, 4D and 4F) showed no distinctive qualitative differences in appearance between each other in proteoglycan or elastic fiber staining. Based on this observation, proteoglycan presence appeared to be equivalent across sample groups treated with BMP7 (Fig 4A and 4B) and qualitatively similar when compared to groups without BMP7 (Fig 3A and 3B). Staining for elastic fibers in BMP7-treated groups (Fig 4E and 4F) may be increased qualitatively in comparison to sample groups without growth factor (Fig 3E and 3F), a result possibly indicative of elevated elastic fiber content in samples putatively enhanced in maturation by BMP7.

**Fig 5:**
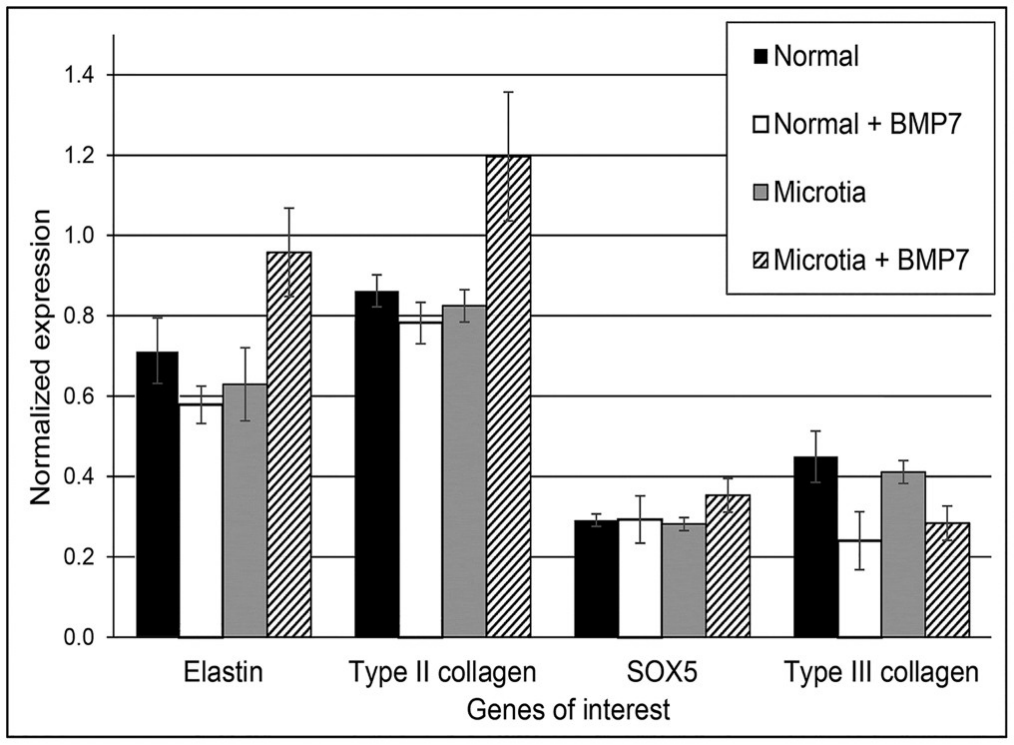
A plot of gene expression values for elastin, type II collagen, SOX5 and type III collagen normalized to large ribosomal fraction, P0.

Fig 5 shows a graphic representation of genes of interest involved in auricular neocartilage ECM generation (type II collagen and elastin) and the maintenance (SOX5) or loss of the cartilage phenotype (type III collagen). Expression level values obtained by qPCR (n = 10 samples for each group, Table 1) were averaged, normalized to the reference gene, P0, and plotted with their respective standard errors of the mean. No statistically significant differences were found for any of the four genes between any of the sample groups. A trend of increasing gene expression of type II collagen, elastin and SOX5 was noted in the BMP7-treated microtia group with a concomitant decrease observed in type III collagen in both BMP7-treated chondrocyte types (Fig 5 and Table 3) when compared to their respective untreated groups, but again the differences in expression values were not statistically significant.

**Table 3:** Trendline equations, R^2^ values and trendline slopes for gene data graphically presented in Fig 5. Calculations were made for trendlines between all groups (four points) and between each biopsy tissue type with and without BMP7 (two points) for each gene of interest.

Gene	Parameter	All groups	Normal and Normal + BMP7	Microtia and Microtia + BMP7
Elastin	Line equation	y = 0.079x + 0.523	y = -0.135x + 0.848	y = 0.329x + 0.301
	R^2^	0.363	1	1
	Slope	Slight positive	Negative	Positive
Type II collagen	Line equation	y = 0.105x + 0.655	y = -0.080x + 0.943	y = 0.372x + 0.453
	R^2^	0.507	1	1
	Slope	Positive	Slight negative	Positive
SOX5	Line equation	y = 0.017x + 0.261	y = 0.002x + 0.290	y = 0.071x + 0.210
	R^2^	0.474	1	1
	Slope	Slight positive	Neutral	Slight positive
Type III collagen	Line equation	y = -0.033x + 0.428	y = -0.209x + 0.659	y = -0.127x + 0.539
	R^2^	0.177	1	1
	Slope	Slight negative	Negative	Negative

Normal (conchal) neocartilage samples with and without BMP7 supplementation were compared with microtia neocartilage with and without BMP7. There were no statistically significant differences between each of the four genes examined within each group of samples. Microtia samples with BMP7 addition showed a trend toward greater expression of elastin, type II collagen and SOX5 compared to all other groups (Table 3, trendline slopes), and both BMP7-enhanced groups exhibited a decreasing trend in type III collagen gene expression when compared with their respective groups without the growth factor (Table 3, trendline slopes). N = 10 for all sample groups. Error bars represent standard error of the mean (SEM) for normalized gene expression values.

Consideration of additional genes and their potential statistical differences in the microtia and normal (conchal) auricular neocartilages whose cells were treated with or without BMP7 supplementation was addressed by microarray analysis of the four sample groups. Results are presented in Tables 1 and 4–6 as follows: Parameters of all samples generated from the surgical tissues utilized in RT-qPCR or microarray analyses or both are given in Table 1; data include sample reference number, patient age and gender, tissue type, subcutaneous implantation site to either the right or left of the mouse spine, and presence or absence of sample supplementation with BMP7. Tables 4–6 identify genes by microarray analysis as statistically significantly different in the tissue-engineered cartilage samples following their retrieval from nude mice after implantation for 10 weeks. Table 4 compares gene expression of cells obtained from otoplasty (normal cartilage) surgeries with and without BMP7, Table 5 compares gene expression of cells from microtia with and without BMP7, and Table 6 compares gene expression of auricular cells from microtia and otoplasty (normal cartilage) surgeries on BMP7 addition to the respective samples. Comparison of gene expression from normal cartilage cells with that of microtia cells, each sample group absent of BMP7 supplementation, yielded no genes that could be detected by microarray analysis as statistically significantly differentially expressed.

**Table 4:** Genes identified by microarray as statistically significantly different in tissue-engineered cartilage. Cells obtained from otoplasty (normal cartilage) surgeries were compared with and without growth factor (BMP7) supplementation and implantation in nude mice for 10 weeks.

Gene Symbol	Gene Name	Gene ID	Fold Change Normal vs Normal + GF	False Discovery Rate Normal vs Normal + GF	Adjusted p*-*value Normal vs Normal + GF
C5orf43	Chromosome 5 open reading frame 43	643155	-3.54	34	0.0164
RFX8	Homo sapiens RFX family member 8	731220	-3.12	34	0.0395
	Chr17:004643132–004643191		-2.68	34	0.0395
TUSC1	Tumor suppressor candidate 1	286319	-2.42	34	0.0395
XLOC_012826	None identified		-2.16	34	0.0395
	ENST00000374945	100996291	-2.04	34	0.0395
TFE3	Transcription factor binding to IGHM enhancer 3	7030	-1.90	34	0.0395
LOC100130938	Uncharacterized LOC100130938		-1.62	34	0.0395

Key: GF = growth factor; Gene ID = number identification in the Gene database of the National Center for Biotechnology Information; RFX = regulatory factor X; IGHM = immunoglobulin heavy constant mu. A negative fold change value indicates downregulation of the normal gene compared to the normal gene with growth factor (GF) supplementation.

**Table 5:** Gene identified by microarray as statistically significantly different in tissue-engineered cartilage. Cells obtained from microtia tissue in reconstruction surgeries were compared with and without growth factor (BMP7) supplementation and implantation in nude mice for 10 weeks.

Gene Symbol	Gene Name	Gene ID	Fold Change Microtia vs Microtia + GF	False Discovery Rate Microtia vs Microtia + GF	Adjusted p*-*value Microtia vs Microtia + GF
ZNF354A	Zinc finger protein 354A	6940	-7.58	76	0.00972

Key: GF = growth factor; Gene ID = identification number in the Gene database of the National Center for Biotechnology Information. A negative fold change value indicates downregulation of the microtia gene compared to the microtia gene with growth factor (GF) supplementation.

**Table 6:** Genes identified by microarray analysis as statistically significantly different in tissue-engineered cartilage. Cells obtained from microtia and otoplasty (normal cartilage) surgeries were compared after growth factor (BMP7) supplementation and implantation in nude mice for 10 weeks.

Gene Symbol	Gene Name	Gene ID	Fold Change Microtia + GF vs Normal + GF	False Discovery Rate Microtia + GF vs Normal + GF	Adjusted p*-*value Microtia + GF vs Normal + GF
ZNF354A	Zinc finger protein 354A	6940	8.82	5.49	0.00139
HOXA10	Homeobox A10	3206	4.76	13.72	0.0174
C5orf43	Chromosome 5 open reading frame 43	643155	-3.41	8.23	0.0119
AVP	Arginine vasopressin	551	3.31	13.72	0.01211
RFX8	Homo sapiens RFX family member 8	731220	-3.15	15.97	0.0174
TUSC1	Tumor suppressor candidate 1	286319	-2.35	15.97	0.03311
KRT85	Keratin 85	3891	-2.32	27.17	0.04526
PABPC3	Poly(A) binding protein, cytoplasmic 3	5042	-2.30	21.78	0.02668
XLOC_012826	None identified		-2.07	31.56	0.04522
TFE3	Transcription factor binding to IGHM enhancer 3	7030	-1.87	15.97	0.03045
XLOC_l2_006821	None identified		1.79	36	0.04526
LOC100130938	Uncharacterized LOC100130938		-1.69	21.04	0.0174
DDX17	DEAD (Asp-Glu-Ala-Asp) box polypeptide 17	10521	1.69	24.39	0.09435

Key: GF = growth factor; Gene ID = number identification in the Gene database of the National Center for Biotechnology Information; RFX = regulatory factor X; IGHM = immunoglobulin heavy constant mu. Negative and positive fold change values respectively indicate downregulation or upregulation of the microtia gene supplemented with growth factor (GF) compared to the counterpart normal gene supplemented with growth factor.

Eight genes [chromosome 5 open reading frame 43 (C5orf43), Homo sapiens RFX family member 8 (RFX8), chr17:004643132–004643191, tumor suppressor candidate 1 (TUSC1), XLOC_012826, ENST00000374945, transcription factor binding to immunoglobulin heavy constant mu (IGHM) enhancer 3 (TFE3), and LOC100130938] were found to be statistically significantly decreased in gene expression levels on comparing normal neocartilage whose cells were treated without and with BMP7 supplementation (Table 4). One gene, zinc finger protein 354A (ZNF354A), was found to be statistically significantly different (decreased) in expression on comparison between microtia neocartilage whose cells were treated without and with BMP7 addition (Table 5). Table 6 presents thirteen genes or related loci that were identified as statistically significantly differentially expressed when microtia-generated neocartilage was compared to normal neocartilage, the respective cells of each group being supplemented with BMP7. Of these thirteen genes, five genes [ZNF354A, homeobox A10 (HOXA10), arginine vasopressin (AVP), XLOC_l2_006821, and DEAD (Asp-Glu-Ala-Asp) box polypeptide 17 (DDX17)] were statistically significantly increased in expression, and eight genes [C5orf43, RFX8, TUSC1, keratin 85 (KRT85), poly(A) binding protein, cytoplasmic 3 (PABPC3), XLOC_012826, TFE3, and uncharacterized LOC100130938 (LOC100130938)] were statistically significantly decreased in expression.

## Discussion

For some time, this laboratory has been interested in examining and characterizing microtia cartilage for its possible application in tissue-engineering reconstruction of an auricle. To that purpose, earlier investigations sought to compare normal (conchal) auricular and microtia tissues in vitro (with and without BMP7) and in vivo, documenting gene expression levels (types II and III collagen, elastin, and SOX5) by RT-qPCR in surgical specimens [33, 34].

Like the present study, previous work utilized chondrocytes from the two tissue types that were seeded onto nPGA sheets identical to those in this report and the cell/scaffold constructs were implanted without BMP7 into nude mice for up to 40 weeks [34]. No qualitative or quantitative differences were found on comparing the sample types in this preliminary analysis [34]. The present work is distinct from its predecessor in that it provides more expansive gene expression findings to determine if microtia surgical remnants may be equivalent to normal auricular cartilage biopsies for their potential autologous auricular reconstruction through tissue engineering. While other investigations have utilized a variety of methods to examine microtia tissue as a surrogate to normal auricular cartilage [21, 24, 25], this study is also the first to apply microarray analysis for that intent. In addition, BMP7, a growth factor known to enhance cartilage matrix production in vitro and in vivo [35, 39] was used as a supplement in these tissue-engineering experiments and analyzed for its effects on the neocartilages developed from the remnants of microtia and normal surgical tissues.

Establishing equivalence between microtia and normal auricular cartilage is not straightforward from an experimental standpoint. Variation in patient samples, differences in experimental protocols and changes in gene expression with donor age and maturation are important factors that must considered in attempting to compare these two types of tissues. To minimize such concerns in the present work, all surgical samples were processed identically from the point of cell isolation, expansion in vitro, seeding onto nanoPGA sheets, implantation in nude mice and harvest. Polymer sheets and BMP7-loaded gelatin beads were prepared in the same manner and analyses of the tissue-engineered four microtia and four normal patient surgical remnants were likewise conducted identically. Morphological dimensional measurements of the retrieved neocartilage specimens, histological presence and content of proteoglycan and elastic fiber content in extracellular matrices, and RT-qPCR of principal genes of interest yielded no significant changes between the two types of auricular neocartilages, engineered on nanoPGA scaffolds in the absence of BMP7. This result was consistent with data obtained from previous preliminary experiments from this laboratory [34]. Comparison of neocartilages using microarray technology that sampled potential quantitative changes in 60 x 10^3^ human genes and analysis with either a restrictive 10% FDR or a less stringent 25% FDR generated no statistically significant differences in any sample genes, again without BMP7 supplementation. The summary qualitative and quantitative results support the concept that human microtia and normal neocartilages are similar and equivalent.

This study also investigated possible cartilage regenerative effects of BMP7 as it was applied to the tissue-engineering protocol. BMP7 is typically used to enhance cartilage extracellular matrix production and prevention of apoptotic or necrotic events with concomitant loss of cartilage phenotype [40]. There were certain observable qualitative differences noted in the presence and content of elastic fibers and cartilage thickness between both neocartilage groups with BMP7 supplementation compared to the respective groups without BMP7 addition with statistical significance noted in thickness comparison between the microtia groups. RT-qPCR analysis showed an increasing trend in gene expression levels of elastin, type II collagen and SOX5 in the microtia group in the presence of BMP7 compared to the other three groups, and both microtia and normal neocartilage groups with BMP7 supplementation were found with decreased type III collagen expression compared to the respective two groups without BMP7 addition. An increase in type III collagen expression over time is indicative of the loss of the cartilage phenotype in chondrogenic cells [28], and the finding of decreased type III expression with BMP7 supplementation to the neocartilages in the constructs fabricated in the present investigation may be suggestive of a protective effect on the cells against such phenotypic changes by this growth factor, a result that has been reported in other studies [35, 40].

With regard to microarray analysis, a 10% FDR yielded no or very few statistically significant differences in gene expression when microtia and normal or BMP7-treated microtia and normal groups were compared respectively. The initial microarray statistical analysis showed a similarity in the neocartilages from microtia or normal surgical biopsies with a low probability of false positives (10% FDR). A change from 10% to 25% FDR increased the number of statistically significant differences in microarray outcomes when both microtia and normal auricular cartilage groups with BMP7 addition were compared to each other and their respective counterpart groups without BMP7 supplementation. The opportunity for detection of false positives was increased in the 25% FDR statistical microarray analysis but the possibility of identifying low abundance genes differentially expressed was also increased and a 25% FDR is not untypical for biological tissues (See S1 File). The addition of the growth factor BMP7 to the microtia- and normal cell-seeded constructs before development in vivo should stimulate cellular growth and proliferation as well as ECM production. Upregulation of genes involved in cellular growth would then be expected on comparison of groups with and without BMP7 addition, but there were unexpected results in addition on comparison involving the microtia cell-seeded constructs with the addition of BMP7. As microarray analysis has not previously been applied to the cells comprising constructs presented in this study, the genes found and statistically significantly differentially expressed are novel and interesting and will be briefly noted in their principal functional aspects in the following paragraphs.

Normal neocartilage generated from conchal cartilage biopsies showed statistically significant increases in expression of eight genes (or loci) when BMP7 was present compared to its absence during construct fabrication prior to their implantation for 10 weeks. Unless otherwise cited, gene functions and descriptions detailed below were obtained from the National Center for Biotechnology Information (ncbi.nlm.nih.gov) Gene Ontology website and GeneCards^®^ Human Gene Database (https://www.genecards.org) websites. Among the eight genes reported in Table 4, these databases identified RFX8 and TFE3 as genes involved in transcriptional control with increased frequency or rate of transcription. More specifically, TFE3 promotes the expression of genes downstream of transforming growth factor beta (TGFβ) signaling, and BMP7 is a known ligand that binds to receptors of the TGFβ superfamily [46]. In the present experiments, BMP7 release from beads led to increasing TFE3 expression, so there may be a dual relation between these two factors in normal neocartilage. TUSC1 function involves cell proliferation, and dysregulation of the TUSC1 gene has been linked to lung cancer (https://www.omim.org).

Four genes (chr17:004643132–004643191, XLOC_012826, ENST00000374945, and uncharacterized LOC100130938) from the same microarray data set have been identified as long non-coding RNAs (lncRNAs). These transcribed lncRNAs do not encode proteins, are the most abundant RNA transcripts, and are different from other non-coding RNAs (such as siRNAs) because their length is greater than 200 nucleotides [47]. Very few lncRNAs have been characterized in detail but research has expanded greatly to examine their function involving roles in many biological processes, including their possible regulation of both activation and inhibition of gene expression [47]. Another interesting aspect of lncRNAs is their ability to interact with membrane proteins like phospholipids and assist in membrane formation [48]. Inhibition, dysregulation or mutation of lncRNAs may result in disease progression and thus many lncRNAs have been associated with several different types of cancer [49]. The presence of these four lncRNAs suggests that they may influence neocartilage generation in the specimens examined in the current study.

The last of the eight genes statistically significantly increased in expression found on microarray analysis comparing normal neocartilage in the presence and absence of BMP7 supplementation is C5orf43. This gene encodes a protein identified as small integral membrane protein 15 (SMIM15), ubiquitous in many tissue types but having limited information available about its function (https://www.ncbi.nlm.nih.gov/pubmed/?term=SMIM15). In the microarray analyses comparing normal-derived neocartilage groups without and with BMP7 supplementation, genes statistically significantly upregulated by the growth factor were found to involve regulation of pathways for transcription, cellular growth and proliferation, and ECM production.

Microarray analysis of the microtia group compared with and without addition of BMP7 yielded only one gene, ZNF354A, statistically significantly differentially expressed. The gene encoding zinc finger protein 354A was almost eight times significantly increased in the BMP7 group when compared to the microtia group without BMP7 addition. Zinc finger protein 354A has DNA-binding transcription factor activity with regulation by RNA polymerase II (www.ncbi.nlm.nih.gov/gene/6940) in relation to the process of sensory perception of sound. Gene ontology defines this process as “the series of events required for an organism to receive an auditory stimulus, convert it to a molecular signal, and recognize and characterize the signal” (http://geneontology.org/GO:0007605). The significance of this finding is unclear as identified for the function of this protein regarding neocartilage formation, but it may somehow be related to auditory processes.

Based on the observation that eight genes were statistically significantly differentially expressed when normal neocartilage was treated with and without BMP7 supplementation, it seems a bit surprising, as mentioned above, that only one gene was identified when microtia cells were treated in the same manner. A possible explanation for only a single gene being identified in the comparison of microtia groups could be related to the microarray analysis itself: Possibly the analysis did not have sufficient power to generate a larger list of genes like that found for normal neocartilage with and without BMP7 addition. In this regard, although each of the experimental specimen groups tested began with a sample number of four, one sample of the microtia group failed its binding to probes during microarray processing and was eliminated from analysis (See Table 1 and S1 File). Therefore, a comparison was made of microtia groups of dissimilar sample numbers, those without (n = 3) and with (n = 4) BMP7 addition. The consequence of these few samples may lead to a decreased power to detect gene differences by microarray analysis. BMP7 addition to microtia cells, compared to the cells without such supplementation, yielded RT-qPCR data that indicated upregulation of ECM genes (type II collagen, elastin and SOX5) and downregulation of type III collagen. Further, microtia cells in the presence compared to the absence of BMP7 showed a quantitative improvement in sample thickness and a qualitative increase in proteoglycan and elastin content based on morphometric and histological analyses, respectively. Thus, there were observable differences in microtia cells with BMP7 compared to the same cells without such supplementation but, again, it was curious that microarray analysis detected only one gene differentially between the two groups. Another possible explanation for only one gene being detected may be that microtia cells respond differently with respect to time to growth factor supplementation compared to that of normal auricular chondrocytes. The upregulation of transcription with BMP7 addition could have occurred early in microtia neocartilage formation and by 10 weeks gene expression levels had stabilized and were not statistically different from microtia cells without BMP7.

Comparison of normal and microtia neocartilage groups with BMP7 supplementation generated a list of thirteen genes or loci statistically significantly differentially expressed. This result was rather unexpected since there were no genes identified in the same manner following comparison of these groups without BMP7 addition. BMP7 present in the gelatin-based delivery system utilized in this study is assumed to be released in a uniform, defined manner over a two- to three-week time frame and thus is equally available to each construct composed of either microtia or normal cells.

An explanation is not immediate for the finding above that several genes were statistically significantly different in expression on comparing normal and microtia tissue supplemented with BMP7. An interesting consideration in this context may be the possibility that different subsets of cells constitute the conchal biopsies and microtia tissue remnants and that BMP7 addition exerts the observed variable effects on such cellular subtypes. The initial surgical samples retrieved for experimental interrogation, for example, were comprised in part of perichondrium whose cells may have been impaired or removed to different degrees during tissue preparation.

The remaining viable perichondrial cells would represent a small population distinct from that of the auricular chondrocyte population and they would vary in their number in each tissue because of the uncertainty in sample treatment. Furthermore, each surgical sample, whether normal or microtic, and the pool of experimental specimens will undoubtedly be different in perichondrial content. In addition, many tissues appear to support resident stem cells that are quiescent in the microenvironment of their ECM and are activated upon injury for the purpose of repair [50, 51]. Indeed, different subsets of tissue-resident stem cells or cartilage stem/progenitor cells have been identified in both auricular perichondrium and auricular cartilage [52–54]. BMP7 has been shown to be active in mediating mesenchymal stem cell recruitment and pathways of chondrogenic maturation and is expressed specifically during the differentiation of stems cells to progenitor cells to chondroblasts [55]. Since gene expression is a constantly changing, orchestrated sequence of events for any cell type, the results noted previously in the thirteen genes following BMP7 addition may reflect different effects, including perhaps those of a temporal nature, exerted by this growth factor on the suggested distinctive subsets of cells populating the normal and microtic tissues of interest.

In this conceptual situation, in which the cells comprising normal and microtic tissues are incompletely defined and the precise effects of BMP7 on these surgical specimens are likewise uncertain, it may be that the gene expression differences found in this study are not so dissimilar.

Therefore, it remains unclear whether long-term outcomes in utilizing chondrocytes from microtia tissue in the presence of BMP7 would lead to any overt effect(s) or result in a microtia-like deformity in auricle reconstruction.

A close examination of the present microarray data reveals six genes (C5orf43, RFX8, TUSC1, XLOC_012826, TFE3, and LOC100130938) that are common and statistically significantly increased on comparison of normal to microtia groups supplemented with BMP7 and of normal cells with and without BMP7. An additional gene that is also statistically significantly increased in normal compared to microtia cells supplemented with BMP7 is PABPC3, a poly(A)-binding protein. PABPs have several roles in mediating gene expression to facilitate messenger RNA stability, translation initiation and termination, and recycling of ribosomes [56]. This set of seven upregulated genes in the comparison of normal and microtia groups of chondrocytes supplemented with BMP7 is involved in pathways for transcription, translation, cellular growth and proliferation. BMP7 addition to normal cells may upregulate several parameters that activate their pathways, such as the TGFβ signaling pathway [46], which possibly are temporally different from those for microtic cells with BMP7 addition. KRT85, a gene also detected as statistically significantly increased in normal compared to microtia cells on supplementation with BMP7, encodes a protein involved in hair and nail formation.

Five genes (ZNF354A, HOXA10, AVP, XLOC_12_006821, and DDX17) were statistically significantly increased on comparison of microtia with normal chondrocytes, each supplemented with BMP7. The function of certain of these molecules, including ZNF354A, has been described more fully above. HOXA10 encodes a DNA-binding transcription factor that regulates gene expression (https://www.genecards.org). The HOX family of genes consists of transcription factors that are critical regulators of embryogenesis, leading to development of highly specific aspects of the body of different species [57]. Mutations in human HOXA1 and HOXA2 are responsible for auricle deformities, involving those of the inner and outer ear, with varying severity [30, 58]. Human HOXA10 is not known to be involved in auricle development, but it targets two genes, TGFβ and FGF2, which may act cooperatively to activate pathways such as β-catenin that are involved in cellular proliferation [59]. AVP, arginine vasopressin, acts as a growth factor by enhancing pH regulation through acid-base transport systems and by positive regulation of cell growth, proliferation and gene expression (https://www.ncbi.nlm.nih.gov/gene/551). XLOC_12_006821 is a lncRNA, but its specific properties have not been fully investigated. However, this lncRNA is related to other examples of lncRNAs, such as XLOC_012826 found in the same and other microarray analyses [47]. Finally, DDX17 mediates cell division and growth through translation initiation and processes involving alteration of RNA secondary structure (https://www.ncbi.nlm.nih.gov/gene/10521). These differentially upregulated genes in the microtia cells with BMP7 compared to normal cells with BMP7 function as promoters of cell proliferation and growth, and their presence may indicate that different pathways or different cellular subtypes as described above are activated in microtia neocartilage formation compared to pathways activated for normal cells supplemented with BMP7 [52–54].

The BMP family of cytokines constitutes part of the TGFβ superfamily and is linked through the SMAD signaling pathway involved in the activation of the HOX gene family [57]. A principal finding of the present work is that BMP7 markedly affects gene expression of HOXA10 as determined by microarray comparison of neocartilages generated from chondrocytes isolated from normal and microtia cartilage surgical remnants. The BMP signaling pathway and the HOX gene family have been implicated to affect mouse auricle morphogenesis with mutations in HOXA2 resulting in microtia [60, 61]. BMP7 is also one of four genes identified as being expressed very early in the otic placode development in chicks [62]. DDX17, a member of the DEAD box proteins implicated in part in cellular growth and division, is one of the principal genes dysregulated in the development of microtia in a pig model by a truncation mutation in HOXA1, and thus DDX17 is also related to the embryogenic development of the microtic auricle [63]. Since there are no embryonic stem cells present in these biopsies, it is unclear as to why these previously unreported genes would be differentially expressed. It may be possible that BMP7 has activated the expression of certain of these early stage genes but without the secretion of the proteins or, again, BMP7 has mediated expression of a particular subset or subsets of auricular cells as discussed above. Further work would be needed to confirm these initial findings related to the HOX and DDX17 genes.

In summary, neocartilage has been generated in vitro from chondrocytes isolated from microtia and normal (conchal) cartilage surgical biopsies and then grown on resorbable nanoPGA fiber mats implanted for 10 weeks in a nude mouse model. The resulting human neocartilages were compared by several methods, including RT-qPCR and, for the first time, microarray analyses to detect possible differences in gene expression. No such differences were found on comparison of the two cell groups but, with addition of the cytokine BMP7 prior to implantation, statistically significant differences between the groups in expression of certain genes were noted by microarray analysis. Most of the gene differences detected in the presence of BMP7 appear related to increasing cellular proliferation and growth through activation of appropriate signaling pathways in both normal and microtia cell groups.

Downstream genes for ECM production and maintenance were examined and type II collagen, elastin and SOX5 gene expression was not statistically significantly different on comparing microarray data for chondrocyte groups with or without BMP supplementation. However, microtia cells treated with BMP7 showed a strong trend toward increased expression of type II collagen and elastin when compared to all other groups. Other gene differences identified between normal and microtia neocartilages following BMP7 supplementation may be related to a suggested possible variability in perichondrial cells remaining after specimen preparation, an undefined cellular composition originating with individual or pooled surgical samples, and/or the normal- and microtia-derived cell population that may contain tissue-resident stem/progenitor chondrocytes. Certain of these genes appear to complement research findings for the BMP and HOX families of genes [57].

In the clinical application of tissue engineering a human ear, it is not known whether the genotypic differences reported in this study between normal and microtia neocartilages, enhanced initially with the growth factor, BMP7, would translate into phenotypic characteristics of microtia with temporal development. There was no indication that patients developed microtic ears in a recent clinical trial in China where tissue-engineered ears were generated from microtia surgical remnants [27]. Almost three years after surgery, this trial reported the appearance of the tissue-engineered ears as normal even while the underlying polymeric scaffold supporting the cells degraded [27]. This clinical investigation did not involve addition of any supplemental growth factor to the tissue-engineered ears before patient implantation.

In contrast to the work just noted, differences between normal and microtia tissue types were discovered in the present study when the chondrocytes were initially prepared with BMP7 and investigated by RT-qPCR and microarray analyses. Here then, the changes do not permit these two isolated pools of cells from surgical biopsies to be considered identical or equivalent in the strictest sense. On the other hand, variations in gene expression of certain ECM proteins were not statistically significant (RT-qPCR) and the FDR underlying microarray study was relaxed, thereby yielding more numerous differentially expressed genes than were detected with a lower FDR value. Only under these circumstances were statistically significantly differentially expressed genes discovered on comparing the cell groups of interest in this work. In this instance, therefore, the present results investigating possible gene expression differences between microtia and normal cells with BMP7 supplementation may be regarded as inconclusive or perhaps circumstantial, particularly with respect to the observations that the same cells in the absence of BMP7 yielded no detectable expression differences. Thus, microtia surgical remnants may be considered as a viable, long-term cell source for auricular reconstructive surgery.

Further, BMP7 may be an important application in microtia-derived neocartilage development and generation, as also occurs with its enhancement of normal chondrocyte proliferation and matrix production determined in this study. More definitive data could be forthcoming from further microarray analyses following experimental implantation times earlier or later than 10 weeks. Additionally, microtia surgical remnants should be compared to normal cartilage, each carefully documented as to its composite cellular subsets, including those derived from the respective intact perichondrium, so that effects of BMP7 and possibly other growth factors can be assessed on the two principal tissues and any other cellular subtypes. Such information would provide greater understanding of the regeneration of auricular cartilage utilizing microtia chondrocytes from surgical remnants and the applicability of using such tissue in engineering a pinna.

## Supporting information

S1 File Microarray analysis final report. The final report outlines the findings of the microarray analyses with explanations of false discovery rate (FDR), statistical packages employed and the attached supporting information (S2–S4 Files).
(PDF)

S2 File Microarray analysis box plots. Quality control (QC) plots designed to determine possible sample problems and array outliers.
(PDF)

S3 File Microarray analysis cluster plots. Hierarchical clustering provides AU (approximately unbiased) p-values as well as BP (bootstrap probability) values computed by means of multiscale bootstrap resampling. Rectangles highlight those clusters with a highly significant p-value (0.05).
(PDF)

S4 File Microarray statistical analysis. Significance analysis of microarrays (SAM).
(PDF)
