# Supplementary material for: An analytical study of neocartilage from microtia and otoplasty surgical remnants: A possible application for BMP7 in microtia development and regeneration
Source: PLoS One. 2020 Jun 17;15(6):e0234650. doi: 10.1371/journal.pone.0234650 (PMC7299323; doi:10.1371/journal.pone.0234650)
Supplement: S1 File — The final report outlines the findings of the microarray analyses with explanations of false discovery rate (FDR), statistical packages employed and the attached supporting information (S2–S4 Files). (PDF) [file pone.0234650.s001.pdf]

NATIONWIDE CHILDREN'S
When your child needs a hospital, everything matters.℠

Statistical Analysis of Agilent Whole Genome Array Data for Robin Jacquet December 17, 2013

This analysis is based on microarrays that were processed in the Biomedical Genomics Core at The Research Institute at Nationwide Children's Hospital, Columbus, Ohio. Sample labeling and hybridizations were performed by *Amy Wetzel* and data analysis by *Peter White* and *Harkness Kuck*. Filenames and samples were as follows:

FileName	BGC ID	Sample ID	Group/ ID	Condition	QC
BGC_253949421902_S01_GE1_1010_Sep10_1_1.txt	1A	709	Female	NormalCartilage	PASS
BGC_253949421902_S01_GE1_1010_Sep10_2_1.txt	2A	866L	Female	NormalCartilage	PASS
BGC_253949421903_S01_GE1_1010_Sep10_1_1.txt	3A	867R	Female	NormalCartilage	PASS
BGC_253949421903_S01_GE1_1010_Sep10_2_1.txt	4A	922R	Male	NormalCartilage	PASS
BGC_253949421902_S01_GE1_1010_Sep10_1_2.txt	5B	748L	Male	MicrotiaCartilage	PASS
BGC_253949421902_S01_GE1_1010_Sep10_2_2.txt	6B	797R	Male	MicrotiaCartilage	FAIL
BGC_253949421903_S01_GE1_1010_Sep10_1_2.txt	7B	813R	Male	MicrotiaCartilage	PASS
BGC_253949421903_S01_GE1_1010_Sep10_2_2.txt	8B	932R	Female	MicrotiaCartilage	PASS
BGC_253949421902_S01_GE1_1010_Sep10_1_3.txt	9A	700L	Female	NormalCartilageGF	PASS
BGC_253949421902_S01_GE1_1010_Sep10_2_3.txt	10A	733L	Male	NormalCartilageGF	PASS
BGC_253949421903_S01_GE1_1010_Sep10_1_3.txt	11A	835L	Female	NormalCartilageGF	PASS
BGC_253949421903_S01_GE1_1010_Sep10_2_3.txt	12A	873L	Female	NormalCartilageGF	PASS
BGC_253949421902_S01_GE1_1010_Sep10_1_4.txt	13B	733R	Male	MicrotiaCartilageGF	PASS
BGC_253949421902_S01_GE1_1010_Sep10_2_4.txt	14B	799L	Male	MicrotiaCartilageGF	PASS
BGC_253949421903_S01_GE1_1010_Sep10_1_4.txt	15B	848L	Male	MicrotiaCartilageGF	PASS
BGC_253949421903_S01_GE1_1010_Sep10_2_4.txt	16B	933L	Female	MicrotiaCartilageGF	PASS

All analysis was performed using analysis scripts that were developed in-house using the R software environment for statistical computing and graphics. These scripts call on several Bioconductor (http://www.bioconductor.org/) packages. Bioconductor is an open source and open development software project to provide tools for the analysis and comprehension of genomic data (Gentleman *et al.*, 2004).

Sample Processing

The concentration of the samples provided was determined using the NanoDrop® ND-1000 UV-Vis Spectrophotometer. RNA samples were analyzed by the BGC using an Agilent 2100 Bioanalyzer Lab-On-A-Chip Agilent 6000 Series II chip to determine the integrity of the samples. The RNA was of high quality and all samples, with the exception of 797R (Microtia Cartilage 3) passed our QC cutoff. Sample labeling and hybridization was performed according to the manufacturer's protocols. **Samples were hybridized to the SurePrint G3 Human GE 8x60K Microarray (AMADID 039494).**

SurePrint G3 Human Gene Expression 8x60K v2 Microarray Kit

Feature	Specification
Agilent Product Number	G4851B
Design ID	039494
Format	8 x 60K
Arrays/Slide	8
Slides/Kit	3
Biological Features	50,599
Replicates of Biological Probes	900 x 10
Positive Controls	96 x 10 ERCC control probes<br>10 x 32 E1A spike-in control probes
Gene List<br>Annotations<br>Design Files<br>Probe Sequences	Visit Agilent's eArray application
Composition	Design based on<br>• RefSeq Build 50<br>• Ensemble Release 52<br>• Unigene Build 216<br>• GenBank (April 2009)<br>• Broad Institute Human lincRNA catalog (Nov 2011)<br>• Broad Institute TUCP transcripts catalog (Nov 2011)
Manufacturing	Agilent 60-mer SurePrint technology

Scanning and Image Analysis

Microarray slides were hybridized overnight, washed and then scanned with Agilent G2505C Microarray Scanner. This high resolution scanner represents the very latest technology from Agilent for Arrays and features an industry-leading extended dynamic range of 10^6 (20-bits) for high sensitivity scanning without saturation, low-level detection resulting from optimized precision optics, broad dynamic range, minimal spectral cross talk that enables detection of weak features. The information about each probe on the array was extracted from the image data using Agilent Feature Extraction 10.10 (FE). This data is stored in the FE ".txt" files. The raw intensity values from these files is imported into the mathematical software package "R", which is used for all data input, diagnostic plots, normalization and quality checking steps of the analysis process using scripts developed in-house specifically for this analysis.

It is very important that you keep a copy of the TXT files as they are required for deposition of array data into repositories such as ArrayExpress or GEO (this has become a requirement for publication in most journals). Furthermore, from these files all of the subsequent analysis can be recreated.

Data Preprocessing

Preprocessing refers to the procedures used to convert raw probe intensities into useful gene expression measurements. For analysis of Agilent Expression data this process consists of

1. Reading in the FE extraction files and extraction the median intensity values for each probe on the array. These intensities are not background corrected (this has been shown to only introduce noise), but are corrected for any scanner offset that was added to the original raw values (see Analysis Log file for details).
2. The dataset is filtered to remove positive control elements and any elements that have been flagged as outliers.
3. Present (P), Marginal (M) or Absent (A) calls are made for each element on the array using in-house methodologies that take into account both the results of FE probe detection statistics and the intensities of the negative controls elements on the array.
4. Data normalization is the process of removing unwanted non-biological variation that might exist between arrays in a microarray experiment. Sources of such variation might include scanner-setting differences, the quantities of mRNA hybridized, processing order and many other factors. Regardless of the normalization approach used, the underlying assumption of the normalization algorithm is that the number of genes changing expression between conditions is relatively small or that an equivalent number of genes increase and decrease in expression. The algorithm for normalization is depending upon whether the design of the experiment was a one-color or two-color approach:
    a. Agilent One-Color Analysis: The median green (Cy3) intensities are normalized between the arrays using the Quantile Normalization package in “R” (Bolstad *et al.*, 2003). Quantile normalization is a non-linear probe-level normalization that results in the same empirical distribution of intensities for each array This is a significantly more robust approach than simply normalizing to the median value of each array.
    b. Agilent Two-Color Analysis: The median red (Cy5) and green (Cy3) intensities are used to calculate the ratio of expression for each element on the array, in terms of M (log2(Red/Green)) and A ((log2(Red) + log2(Green))/2)). The M values are then global loess normalized within each array using the LIMMA microarray processing package in “R” (Smyth, 2004). This intensity based normalization approach adjusts the red and green intensities relative to one another so that the red/green ratios are as far as possible an unbiased representation of relative expression.

Quality Control Diagnostics

A frequently overlooked, yet critically important, additional step to data preprocessing is the ability to distinguish between good and poor quality arrays in a given data set. This is done by visualizing the data before and after the various preprocessing steps and through the generation of quality control (QC) metrics. In addition to the Agilent FE QC reports (see PDF files) we

generate several QC plots that are designed to highlight outlying arrays or potential issues with samples.

Intensity Distribution Plots. One of the underlying assumptions for the normalization procedure is that the probe data all come from the same distribution, with the only differences being the location and scale. Basically, in the *Intensity Histogram* plots this means that we want the shape of the curves to be very similar, and we want the curves to be fairly close to each other.

Another useful visualization tool to display the distribution of the data is through the use of box plots. These plots are used to graphically depict numerical data using their five-number summaries: the sample minimum, lower quartile (Q1), median (Q2), upper quartile (Q3) and the sample maximum. Any observation that lies more than 1.5 x IQR lower than Q1 or higher than Q3 is considered an outlier, as indicated by the "whiskers". As such, they allow us to view the distribution of raw expression values and identify any arrays that are outliers. Furthermore, after normalization the distribution of probe intensities should be the same for all the arrays.

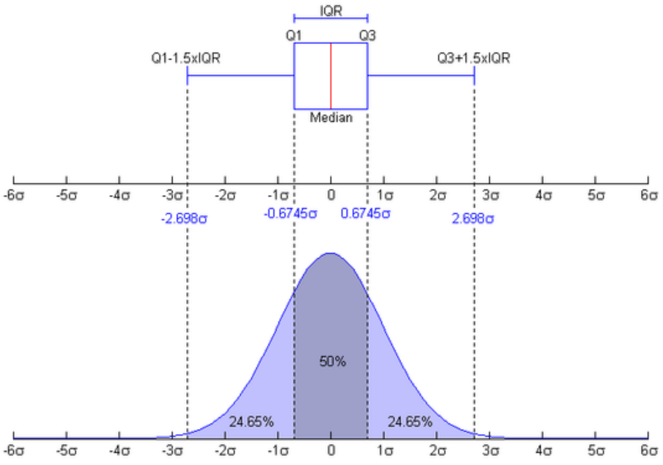

RLE Plots. Another quality assessment tool are Relative Log Expression (RLE) values. Specifically, these RLE values are computed for each probe set by comparing the expression value on each array against the median expression value for that probe set across all arrays. Assuming that most genes are not changing in expression across arrays means ideally most of these RLE values will be near 0. Boxplots of these values, for each array, provides a quality assessment tool. When examining this plot focus should be on the shape and position of each of the boxes. Typically arrays with poorer quality show up with boxes that are not centered about 0 and/or are more spread out. ***For this particular experiment, all array quality control metrics, with the exception of 797R (Microtia Cartilage 3), met or exceeded our quality control expectations. Sample 797R did not label well, and resulting array intensity was below standard. To minimize the impact of this outlier on the analysis, this sample was removed from all subsequent analysis steps.***

Statistical Analysis

Microarrays measure the expression of thousands of genes to identify changes in expression between different biological states. As such, methods are needed to determine the significance of these changes while accounting for the enormous number of genes tested. One significant

challenge inherent to high-throughput analysis of large scale changes in gene expression is the development of statistical methods that will maximize both the sensitivity and specificity of detection of differentially expressed genes. Although several tools are available, we focus on two well validated and commonly utilized approaches to perform our statistical analysis:

Significance Analysis of Microarrays (SAM). SAM is a powerful tool for analyzing microarray gene expression data useful for identifying differentially expressed genes between two conditions (Tusher *et al.*, 2001). SAM calculates a test statistic for relative difference in gene expression based on permutation analysis of expression data and calculates a false discovery rate (FDR) using the q-value method presented in Storey (2003). In outline, SAM identifies statistically significant genes by carrying out gene specific t-tests and computes a statistic for each gene, which measures the strength of the relationship between gene expression and a response variable. This analysis uses non-parametric statistics, since the data may not follow a normal distribution. The response variable describes and groups the data based on experimental conditions. In this method, repeated permutations of the data are used to determine if the expression of any gene is significant related to the response. The use of permutation-based analysis accounts for correlations in genes and avoids parametric assumptions about the distribution of individual genes. For this experiment, SAM analysis was implemented in R using the Bioconductor Siggenes package. In a one-color experimental design a *two-class unpaired* analysis is typically performed for each experimental comparison, whereas in a two-color approach a *one-class* analysis is used. Typically, an FDR cutoff in the range of 10-20% is chosen to maximize sensitivity without significantly impacting accuracy. ***For the current study, a 25% FDR was used to generate the list of significantly differentially expressed genes. The q-values (FDR) for each gene are provided in the results table – the lower the value the more significant the result. A standard FDR would be 10%, but due to the high level of biological variation in these samples, very few genes met this stringent cutoff. This is not uncommon with experiments performed using primary human tissues.***

Adjusted p-value. The moderated t-statistic (t) is computed for each probe and for each contrast in the experimental design. This has the same interpretation as an ordinary t-statistic except that the standard errors have been moderated across genes, i.e., shrunk towards a common value (Smyth, 2004). This has the effect of borrowing information from the ensemble of genes to aid with inference about each individual gene. The p-value (p-value) is obtained from the distribution of the moderated t-statistic. Finally these p-values are adjusted for multiple testing using the Benjamini and Hochberg's (1995) step-up method for controlling the false discovery rate. This is the most popular method for p-value adjustment. If all genes with p-value below a threshold, say 0.05, are selected as differentially expressed, then the expected proportion of false discoveries in the selected group is controlled to be less than the threshold value, in this case 5%. ***For the current study, the adjusted p-values were calculated using the Bioconductor limma package.***

Although we provide results using both methods, our recommendation is that one should initially focus on the gene list generated with the SAM FDR statistic, as this is often the most commonly reported. Furthermore, it can be considered to more robust as it is a non-

parametric approach and does not assume equal variance and/or independence of genes. Please note that prior to statistical analysis, all control probes were filtered out of the dataset.

Cluster Analysis

Hierarchical clustering was performed on the samples (arrays) using the “R” package “pvclust” (Suzuki and Shimodaira, 2006). This package calculates p-values for hierarchical clustering via multiscale bootstrap resampling. Hierarchical clustering is done for given data and p-values are computed for each of the clusters. It provides AU (approximately unbiased) p-values as well as BP (bootstrap probability) values computed via multiscale bootstrap resampling. The cluster dendrogram highlights AU p-values (printed in red) and BP values (printed in green), which is less accurate than AU value as p-value. One can consider that clusters (edges) with high AU values (e.g. 95%) are strongly supported by the data. Rectangles highlight those clusters with a highly significant P value (0.05), significant clusters within these highlighted clusters are not highlighted. These plots can be found in the RAnalysis/Cluster_Plots directory.

References

Benjamini, Y. and Y. Hochberg (1995). "Controlling the false discovery rate: a practical and powerful approach to multiple testing." Journal of the Royal Statistical Society, Series B 57: 289–300.

Bolstad, B. M., R. A. Irizarry, et al. (2003). "A comparison of normalization methods for high density oligonucleotide array data based on variance and bias." Bioinformatics 19(2): 185-93.

Gentleman, R. C., V. J. Carey, et al. (2004). "Bioconductor: open software development for computational biology and bioinformatics." Genome Biol 5(10): R80.

Smyth, G. K. (2004). "Linear models and empirical bayes methods for assessing differential expression in microarray experiments." Stat Appl Genet Mol Biol 3: Article3.

Storey, J. D. and R. Tibshirani (2003). "Statistical significance for genomewide studies." Proc Natl Acad Sci U S A 100(16): 9440-5.

Suzuki, R. and H. Shimodaira (2006). "Pvclust: an R package for assessing the uncertainty in hierarchical clustering." Bioinformatics 22(12): 1540-2.

Tusher, V. G., R. Tibshirani, et al. (2001). "Significance analysis of microarrays applied to the ionizing radiation response." Proc Natl Acad Sci U S A 98(9): 5116-21.

Wu, Z. and R. A. Irizarry (2005). "Stochastic models inspired by hybridization theory for short oligonucleotide arrays." J Comput Biol 12(6): 882-93.

Microsoft Excel has a powerful feature that can help make shorter lists from this long list by allowing the user to apply filters to the data. On the Data menu, point to Filter, and then click AutoFilter. AutoFilter arrows appear to the right of each column heading. Click an AutoFilter arrow and then click (Custom…) to open the Custom AutoFilter dialog box. For example, using this feature you can select all genes with a Fold Change value greater than or equal to 1.5. As an example of what we typically use in the Functional Genomics Core, the following two worksheets were generated using custom filters. A very useful tutorial for this feature of Excel can be viewed at:

http://office.microsoft.com/training/training.aspx?AssetID=RC011459671033
