# Supplementary material for: An analytical study of neocartilage from microtia and otoplasty surgical remnants: A possible application for BMP7 in microtia development and regeneration
Source: PLoS One. 2020 Jun 17;15(6):e0234650. doi: 10.1371/journal.pone.0234650 (PMC7299323; doi:10.1371/journal.pone.0234650)
Supplement: S2 File — Quality control (QC) plots designed to determine possible sample problems and array outliers. (PDF) [file pone.0234650.s002.pdf]

S2 File. Quality control (QC) box plots to determine sample integrity and to identify array outliers.

Key for array, green channel and relative expression box plots: Red = normal cartilage, green = microtia cartilage, blue = normal cartilage + BMP7, yellow = microtia cartilage + BMP7.

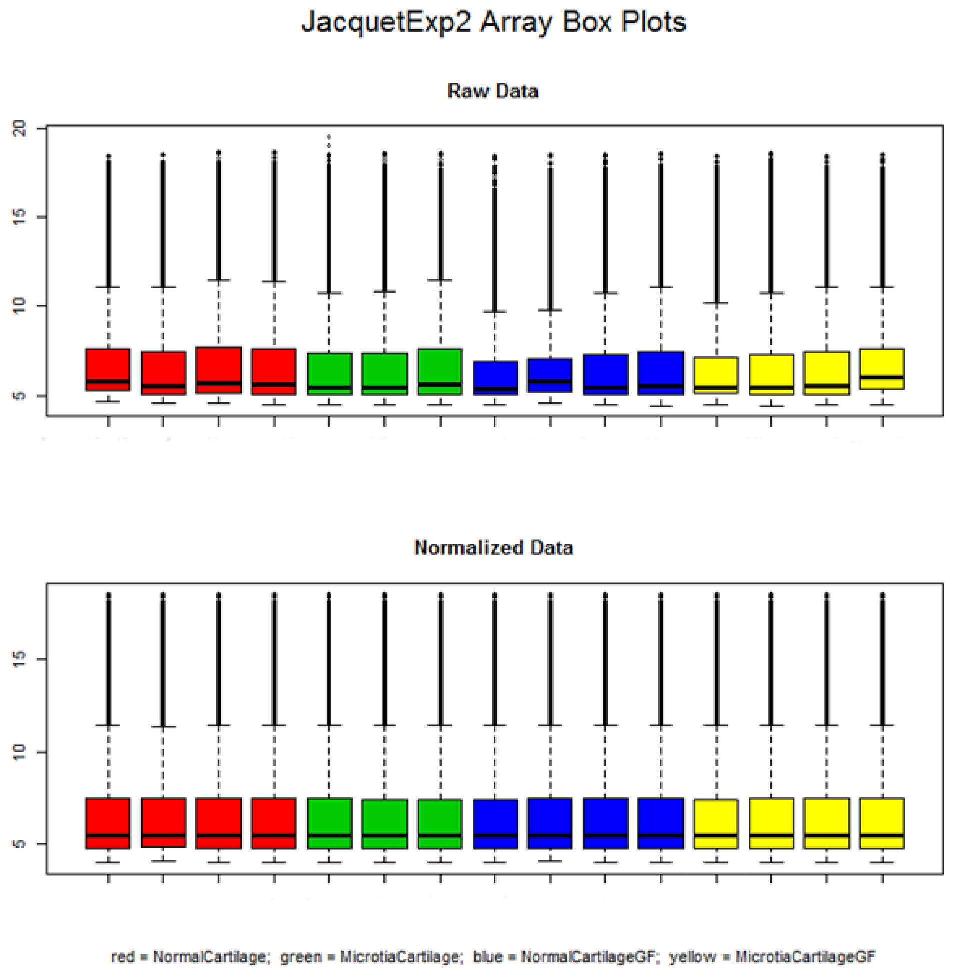

Green Channel Background

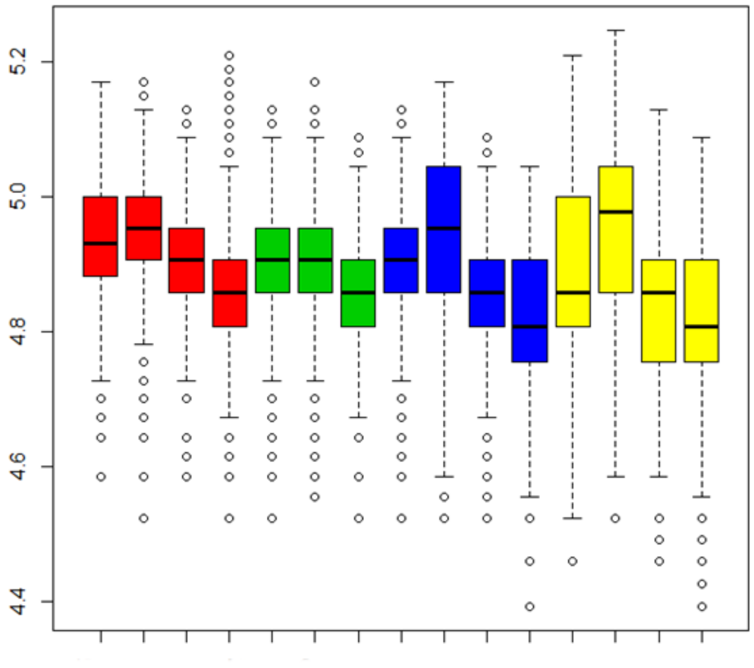

Relative Log Expression: JacquetExp2

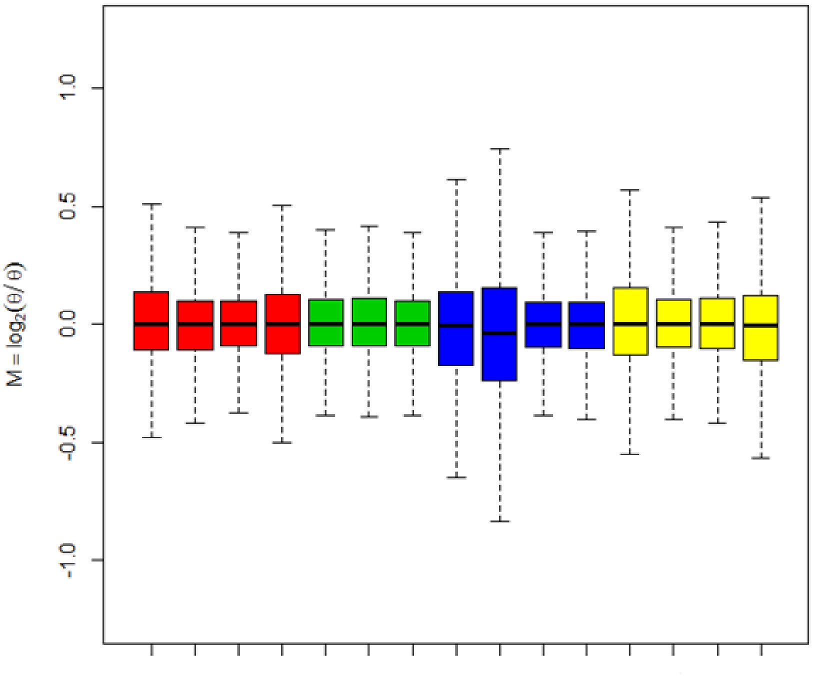

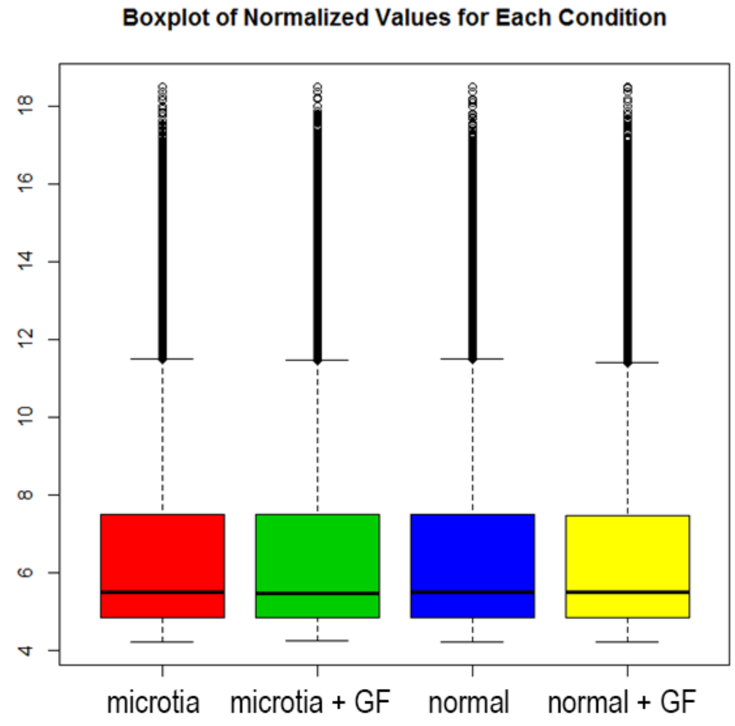

Boxplot of Normalized Values for Each Condition
4
6
8
10
12
14
16
18
microtia
microtia + GF
normal
normal + GF
