# Supplementary material for: An analytical study of neocartilage from microtia and otoplasty surgical remnants: A possible application for BMP7 in microtia development and regeneration
Source: PLoS One. 2020 Jun 17;15(6):e0234650. doi: 10.1371/journal.pone.0234650 (PMC7299323; doi:10.1371/journal.pone.0234650)

S3 File. Microarray analysis cluster plots.

The cluster dendrogram highlights AU p-values (printed in red) and BP values (printed in green), which are less accurate than AU values as p-values. One can consider that clusters (edges) with high AU values (for example, 95%) are strongly supported by the data. Rectangles highlight those clusters with a highly significant p-value (0.05); significant clusters within these highlighted clusters are not highlighted.

1. Cluster diagram representing all probes fold change

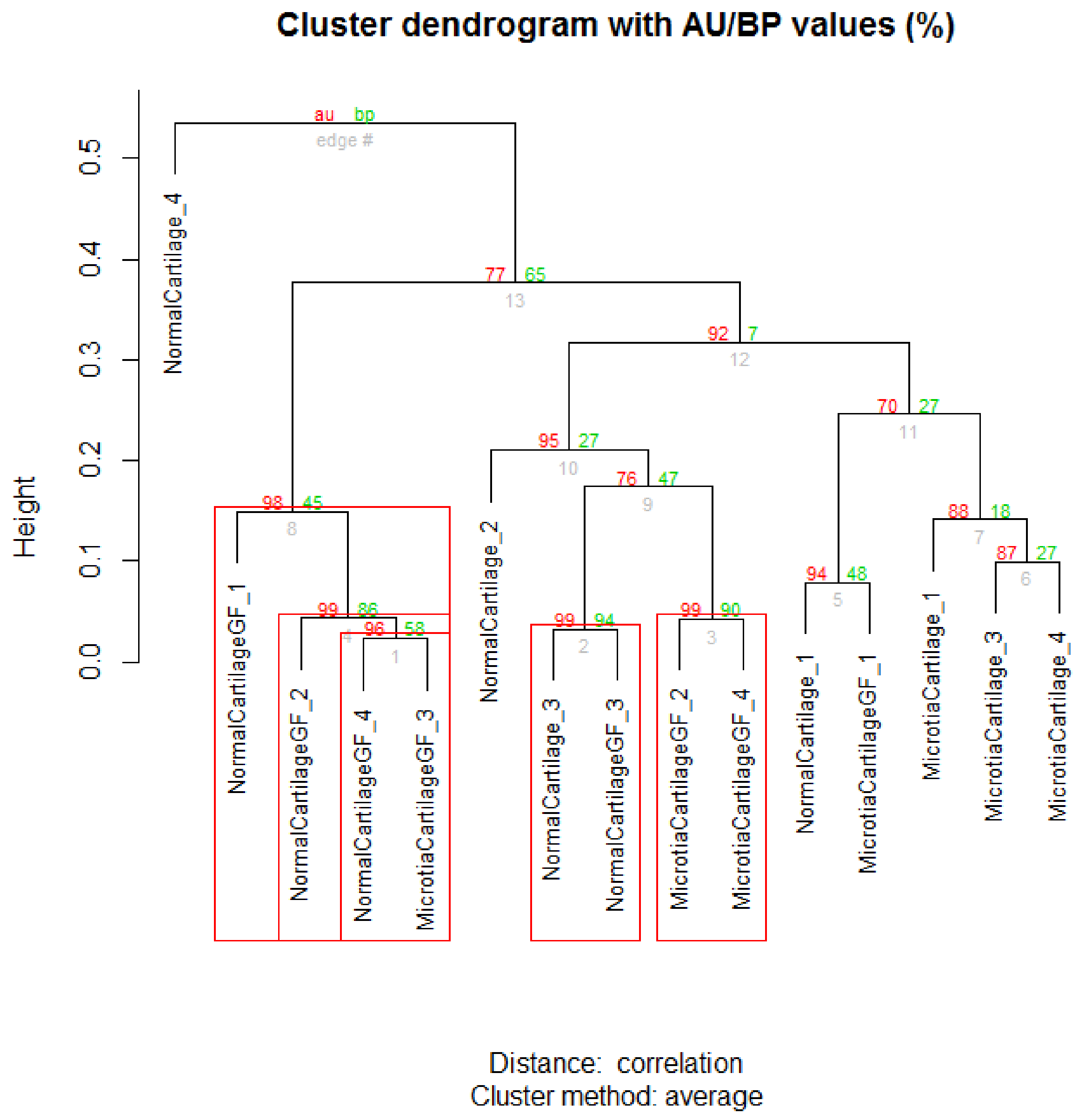

2. **Cluster diagram representing all probes fold SD filtered**

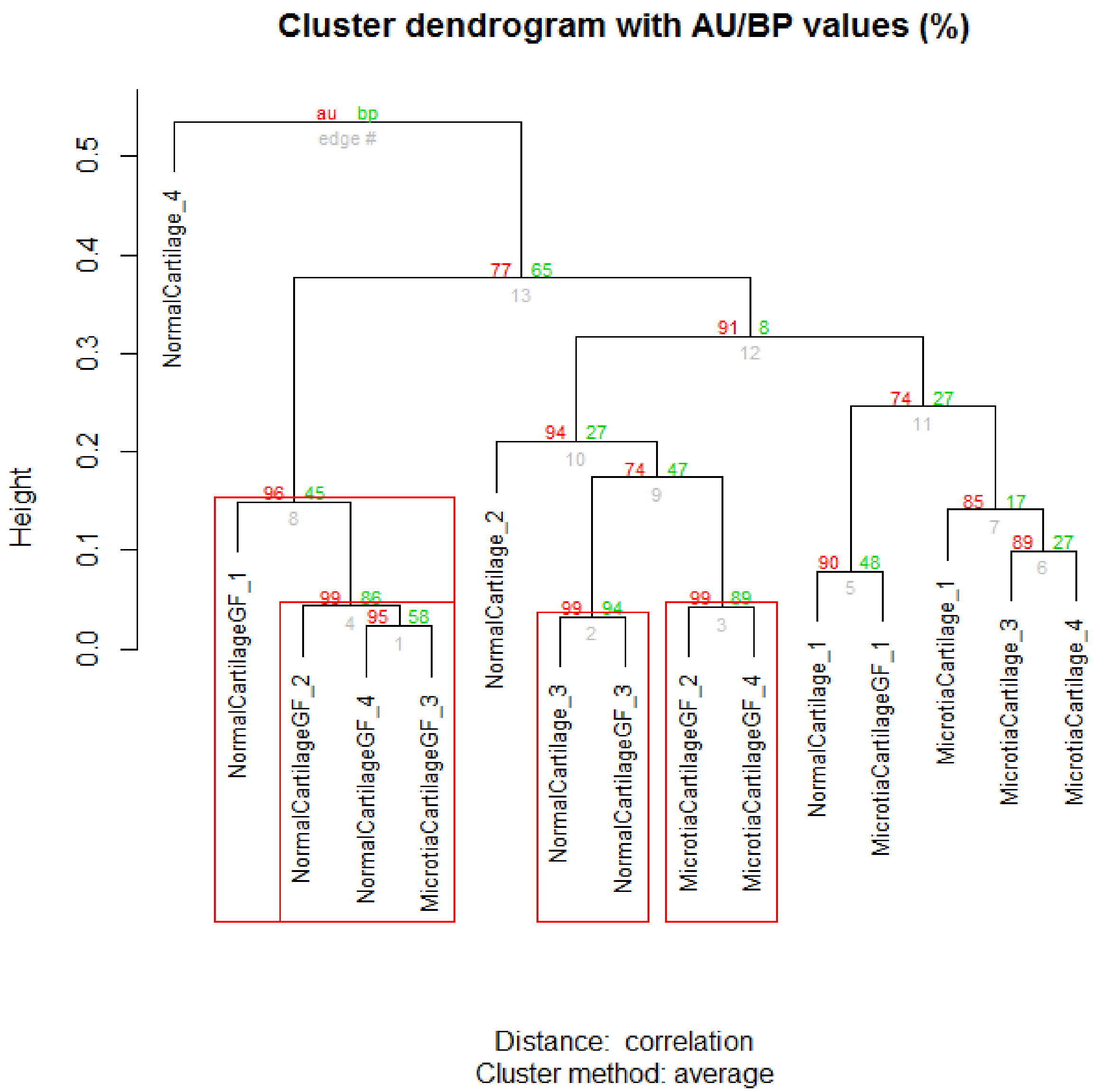

3. Cluster diagram representing all probes

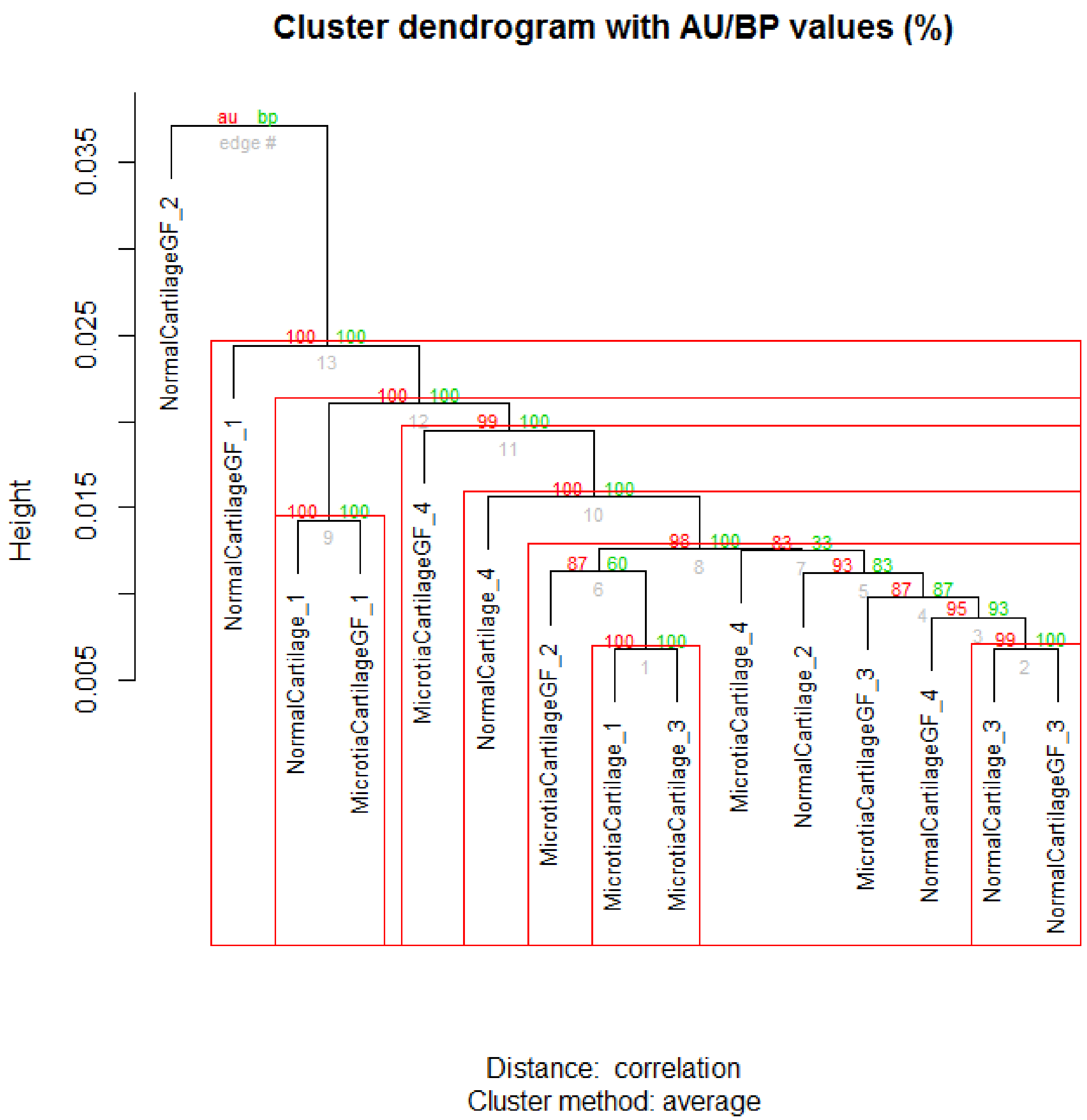

4. **Cluster diagram representing SD filtered**

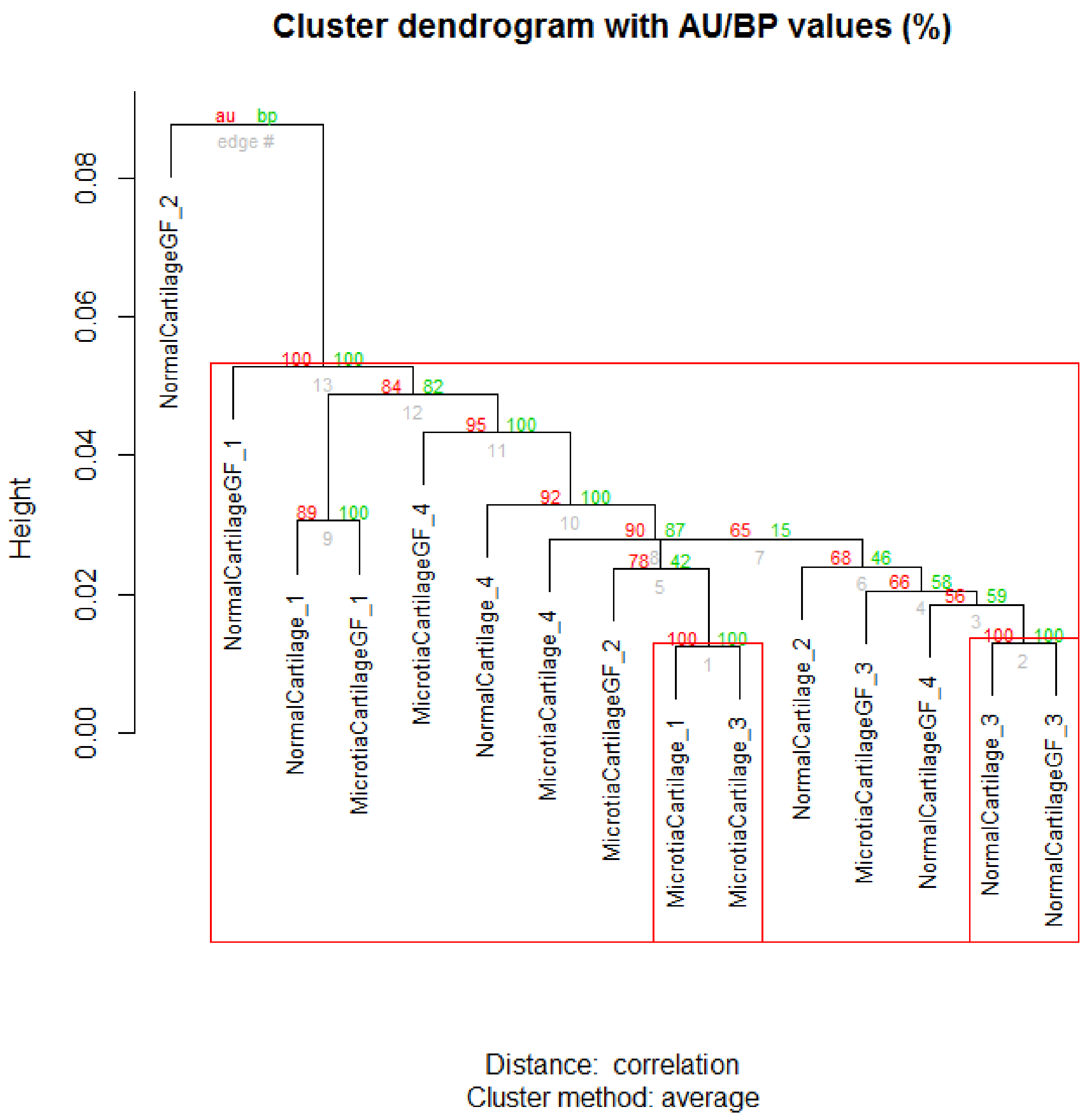

Supplement: S3 File — Hierarchical clustering provides AU (approximately unbiased) p-values as well as BP (bootstrap probability) values computed by means of multiscale bootstrap resampling. Rectangles highlight those clusters with a highly significant p-value (0.05). (PDF) [file pone.0234650.s003.pdf]
