# Supplementary material for: An analytical study of neocartilage from microtia and otoplasty surgical remnants: A possible application for BMP7 in microtia development and regeneration
Source: PLoS One. 2020 Jun 17;15(6):e0234650. doi: 10.1371/journal.pone.0234650 (PMC7299323; doi:10.1371/journal.pone.0234650)

S4 File. Microarray statistical analysis. Significance analysis of microarrays (SAM).

Group: Microtia cartilage and normal cartilage without BMP7

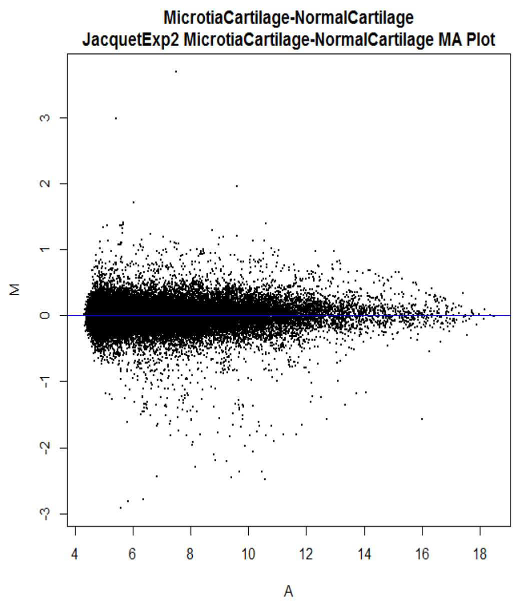

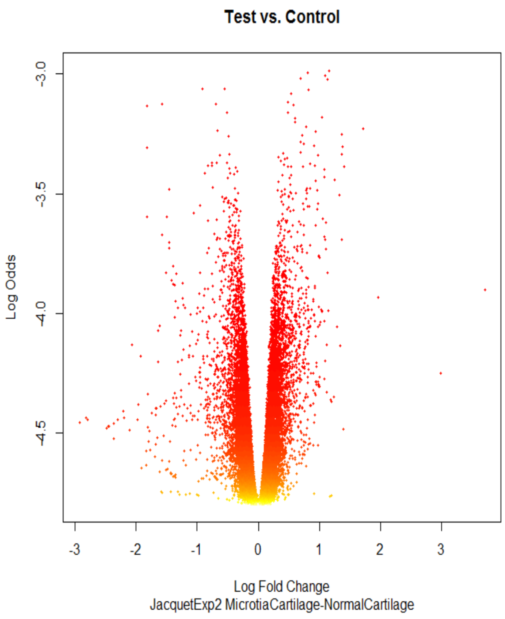

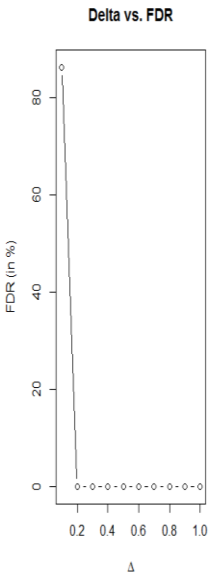

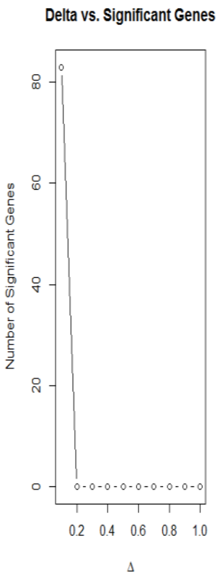

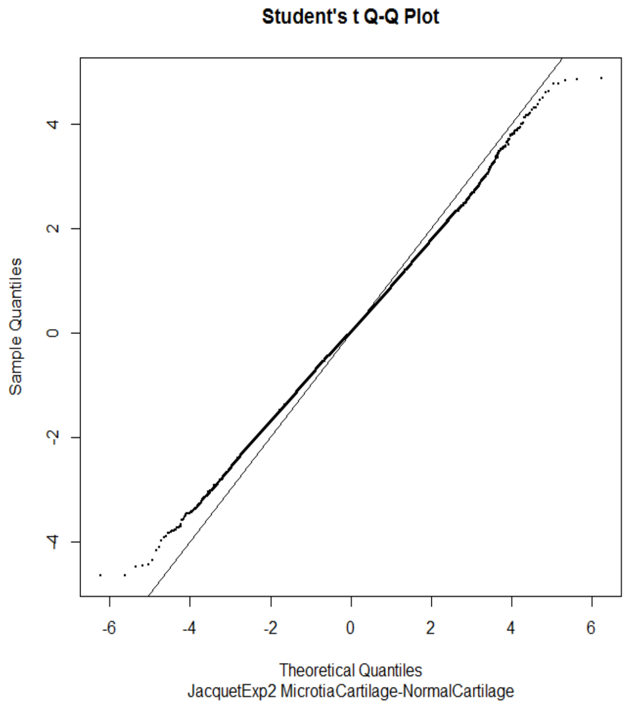

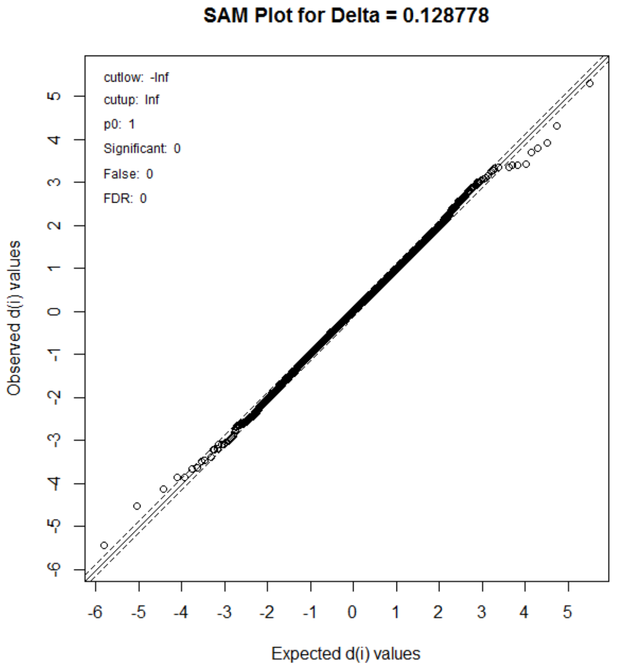

Group: Microtia cartilage and normal cartilage with BMP7

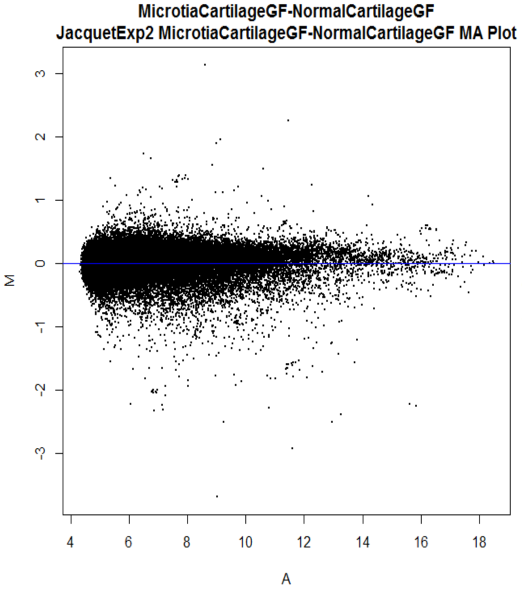

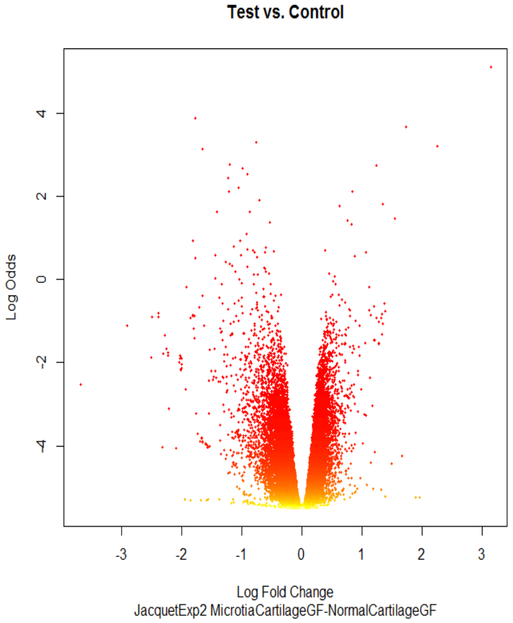

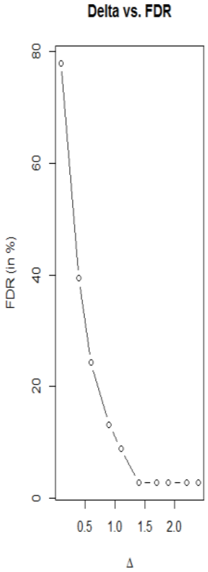

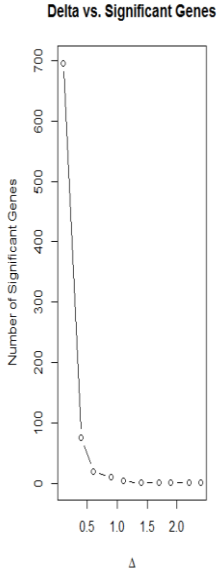

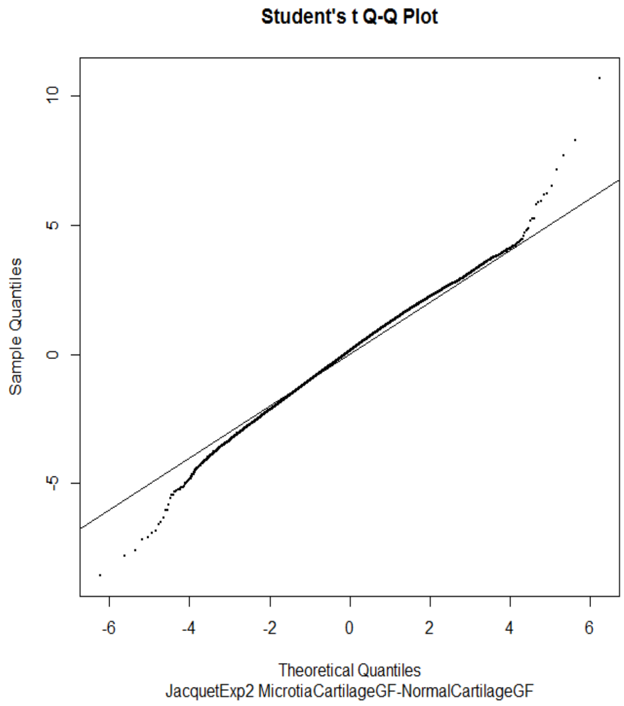

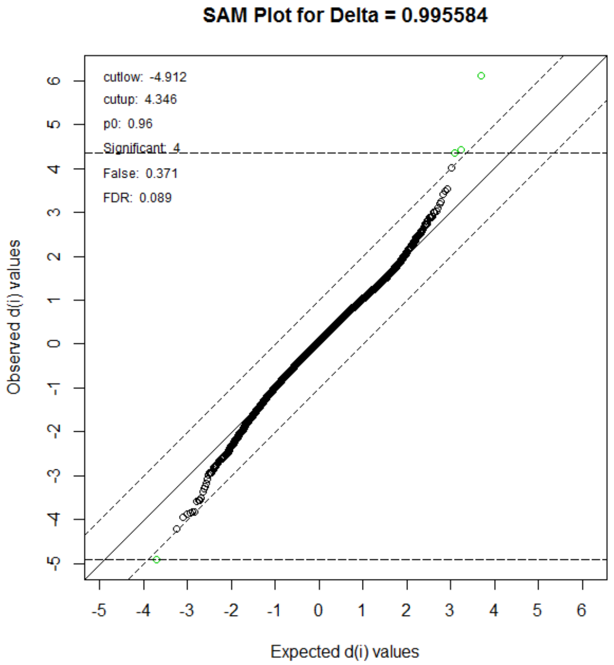

Group: Microtia cartilage with and without BMP7

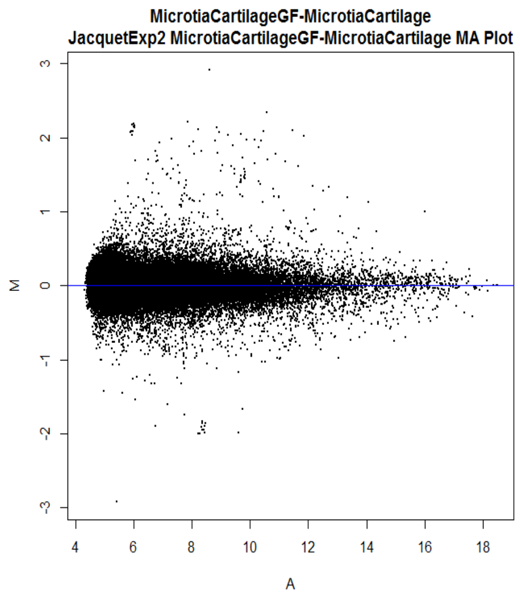

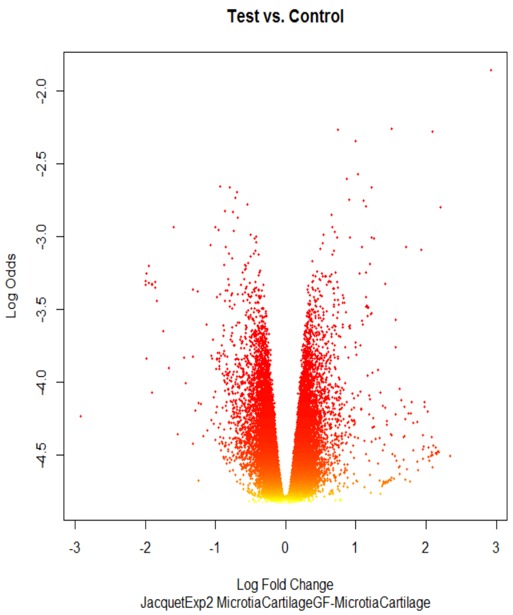

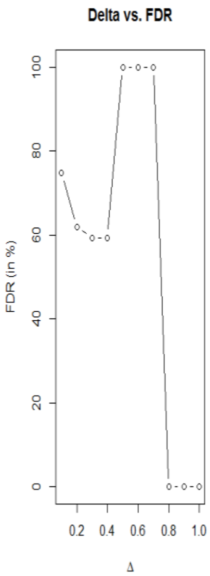

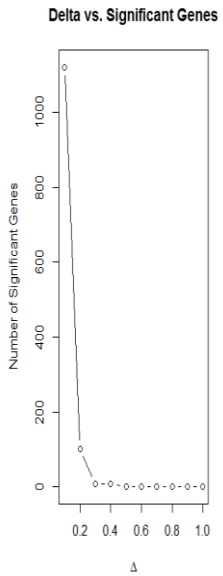

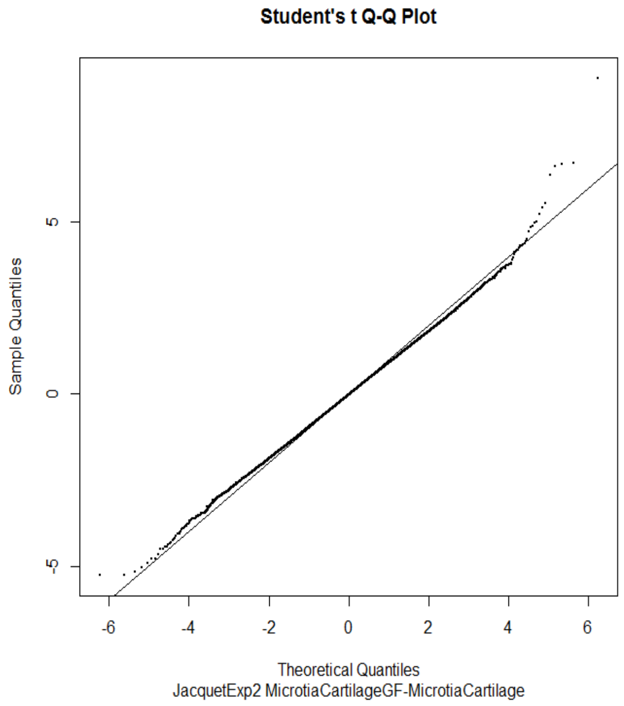

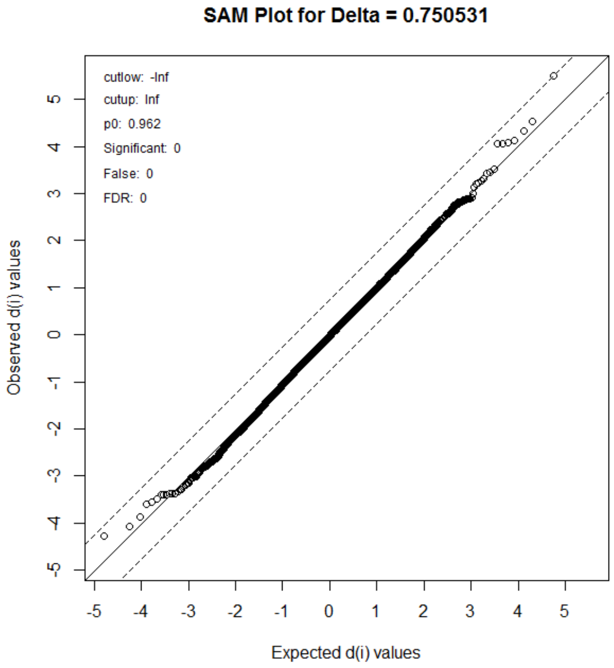

Group: Normal cartilage with and without BMP7

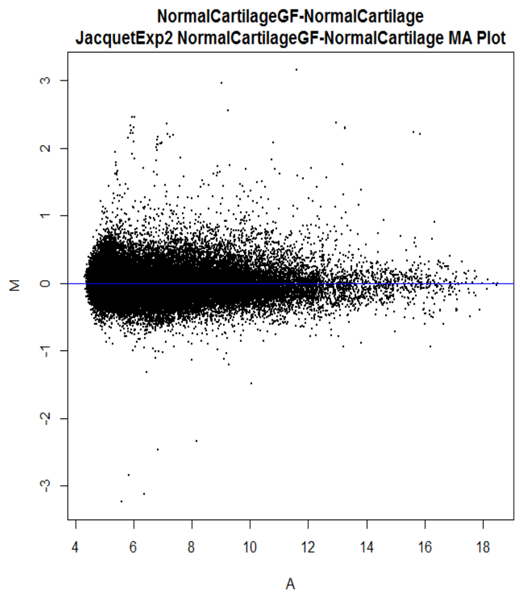

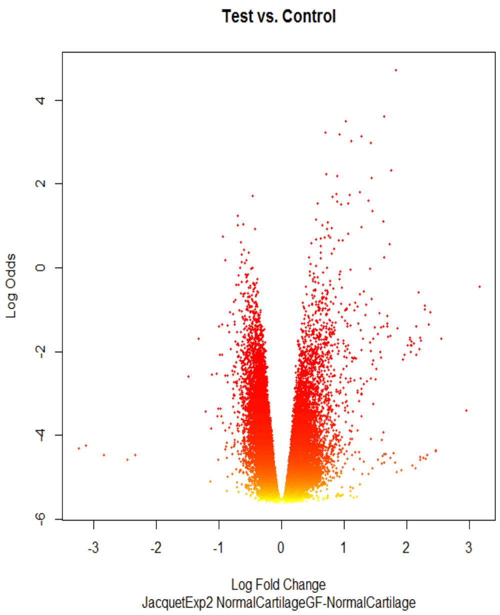

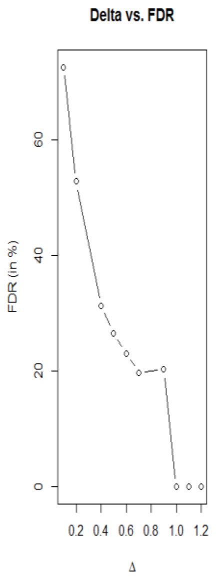

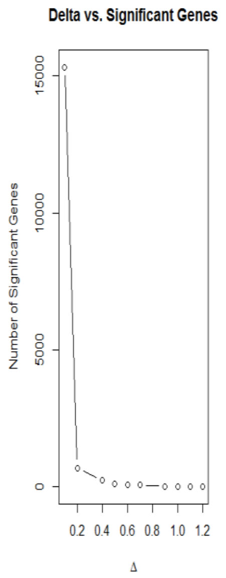

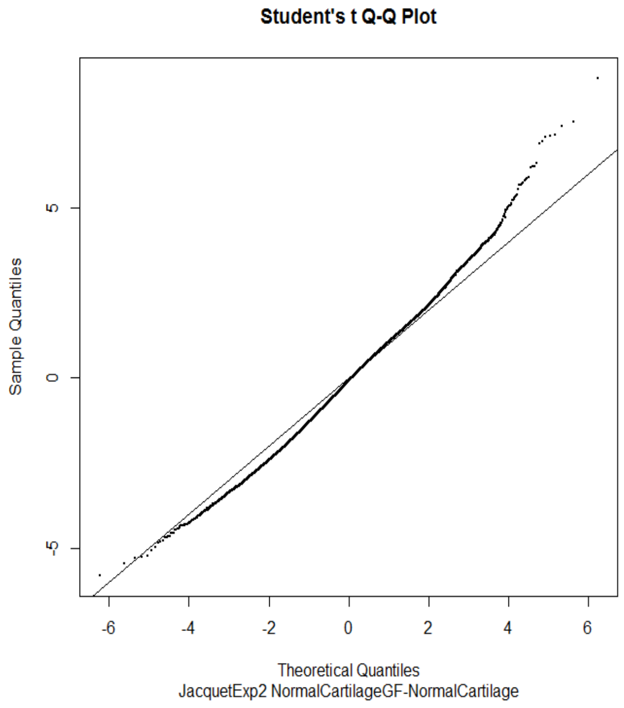

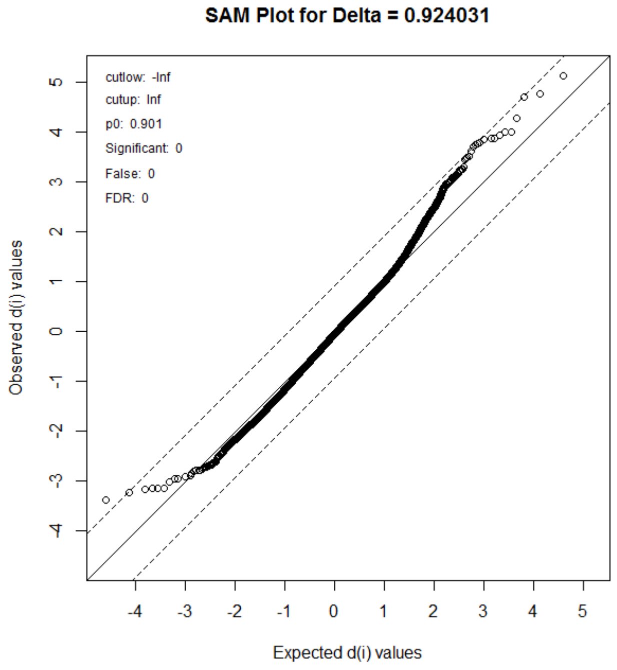

Supplement: S4 File — Significance analysis of microarrays (SAM). (PDF) [file pone.0234650.s004.pdf]
